# Meltable, Glass-Forming, Iron Zeolitic Imidazolate Frameworks

**DOI:** 10.1021/jacs.3c01455

**Published:** 2023-05-09

**Authors:** Luis León-Alcaide, Rasmus S. Christensen, David A. Keen, José L. Jordá, Isaac Brotons-Alcázar, Alicia Forment-Aliaga, Guillermo Mínguez Espallargas

**Affiliations:** † Instituto de Ciencia Molecular (ICMol), Universidad de Valencia, c/ Catedrático José Beltrán, 2, 46980 Paterna, Spain; ‡ ISIS Facility, Rutherford Appleton Laboratory, Harwell Campus, Didcot, Oxfordshire OX11 0QX, U.K.; § Center for Integrated Materials Research, Department of Chemistry and iNANO, Aarhus University, Langelandsgade 140, 8000 Aarhus C, Denmark; ∥ Instituto de Tecnología Química (UPV-CSIC), Universitat Politècnica de València−Consejo Superior de Investigaciones Científicas, Av. de los Naranjos s/n, 46022 Valencia, Spain

## Abstract

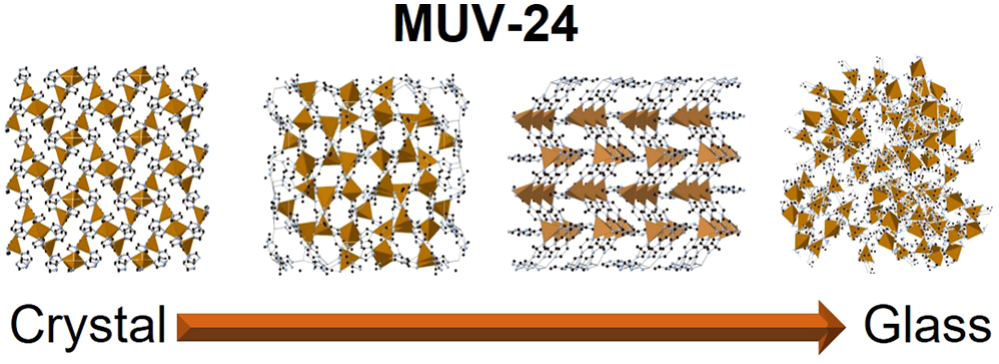

We describe the first meltable iron-based zeolitic imidazolate framework (ZIF), denoted **MUV-24**. This material, elusive from direct synthesis, is obtained from the thermal treatment of [Fe_3_(im)_6_(Him)_2_], which yields Fe(im)_2_ upon loss of the neutral imidazole molecules. Different crystalline phase transformations are observed upon further heating, until the material melts at 482 °C. Vitrification upon cooling of the liquid phase gives rise to the first Fe-metal–organic framework glass. X-ray total scattering experiments show that the tetrahedral environment of the crystalline solids is maintained in the glass, whereas nanoindentation measurements reveal an increase in Young’s modulus, in agreement with stiffening upon vitrification.

## Introduction

Crystalline metal–organic frameworks (MOFs) are currently one of the most studied classes of materials, although these porous materials typically collapse on heating under irreversible decomposition.^1−3^ However, the combination of thermal stability with suitable functionalization has led to the appearance in these materials of a molten phase prior to decomposition that was unnoticed until recently.^4−7^ MOF glasses are generated on cooling these melts.^8,9^ Both MOF liquids and glasses have recently gained much interest because of their uncommon physical properties such as different mechanical properties,^4,10,11^ porosity,^12,13^ ionic conductivity,^14,15^ or application in devices (e.g., as electrolytes,^16^ in solar cells,^17^ as membranes,^18^ anodes for Li-ion batteries^19^).

Despite the large number of MOFs already reported (more than 100,000 in the CCDC),^20^ those that melt prior to decomposition are very scarce. Specifically, these mainly belong to the family of zeolitic imidazolate frameworks (ZIFs),^21,22^ which are formed by M^2+^ cations linked by imidazolate derivative anions. Only a handful of Zn^2+^ and Co^2+^ based ZIFs have been reported to melt and form hybrid glasses, namely, ZIF-4^8b^ and ZIF-62(Zn and Co),^10,23,24^ all containing imidazolate bridges (im^–^).

Reducing the melting temperature is very desirable, as it would reduce the energy required for the formation of the glasses and facilitate its interaction with other materials.^5,25,26^ The most used strategy to achieve this reduction has been the incorporation of bulkier ligands.^8a,11,13,27−31^ In this sense, incorporating small amounts of bulkier imidazole derivatives, such as benzimidazole, reduces the melting temperature (*T*_m_) from 590 °C (as found in Zn(im)_2_, also known as ZIF-4),^8b^ to 310 °C (as found in Zn_0.8_Co_0.2_(im)_1.95_(bim)_0.025_(Clbim)_0.025_).^31^ In addition, it is also very important to increase the temperature interval between melting and thermal decomposition, in order to facilitate the preparation of glasses. This has also been achieved using bulky substituents, with the largest reported interval of approximately 200 °C for ZIF-62 derivates.^8a^

In this work, motivated by the prospect of expanding the family of ZIF glasses, we explore the preparation of a novel Fe-glass based on Fe(im)_2_, through an indirect manner, as this compound is unachievable by a direct synthetic route. This will also allow the exploration of the effects on *T*_m_ of incorporating the more labile iron(II) centers.

## Results and Discussion

The nonporous coordination polymer [Fe_3_(im)_6_(Him)_2_] (CCDC code = **IMIDFE**) was prepared, as previously reported,^32^ in a solvent-free reaction that also serves to prepare porous Fe-based ZIFs.^33,34^**IMIDFE** consists of a 3D coordination polymer with alternating tetrahedral and octahedral centers which are linked via imidazolate bridges. Each iron connects to four other metals, with the octahedral centers having two terminal imidazole molecules in *trans*-configuration.

**Figure 1:**
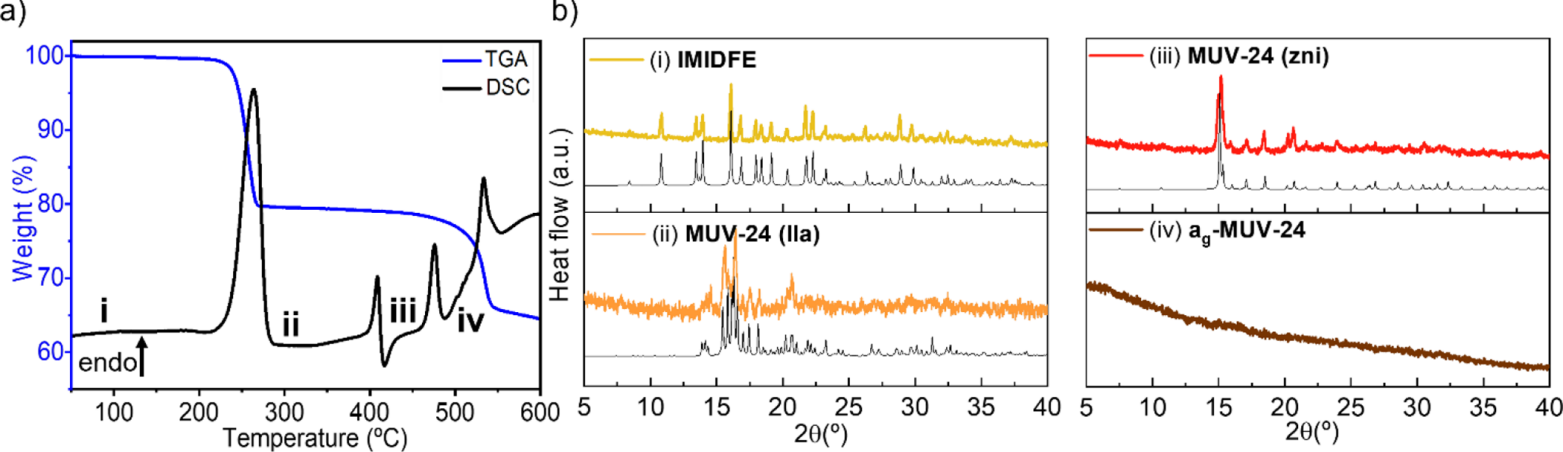
(a) Thermogravimetric (in blue) and differential scanning calorimetry (in black) analyses upon heating [Fe_3_(im)_6_(Him)_2_] (**IMIDFE**), showing the mass loss and the phase transitions; (b) X-ray powder patterns of the different phases obtained upon heating at different temperatures (indicated in the DSC plot), showing the calculated powder patterns as thin black lines at the bottom.

Upon heating this solid, a mass loss of 21.3% is observed in TGA (under a N_2_ atmosphere) at ca. 283 °C, which is accompanied by an endothermic peak in the DSC measurement (see Figure 1a). This mass loss can be associated with the removal of the terminal imidazole molecules (calc. 19.3%), which is confirmed by TGA coupled to mass spectrometry (see Figure S21). This process is accompanied by a change in the powder X-ray pattern (Figure 1b), revealing a rearrangement in the structure of the solid with formula Fe(im)_2_. Fortunately, a small single crystal could be isolated upon heating **IMIDFE** at 300 °C, thus allowing the unequivocal identification of structural changes. The new solid, denoted **MUV-24(lla)**, was solved from single crystal X-ray diffraction and revealed a new polymorph of Fe(im)_2_ with a hitherto unknown topology. This new phase crystallizes in the space group *P*2_1_/*c* (*a* = 12.358 Å, *b* = 23.643 Å, *c* = 19.556 Å, β = 93.237 °) with a 3D structure formed only of tetrahedral Fe(II) centers coordinated via imidazolate ligands.

**Figure 2:**
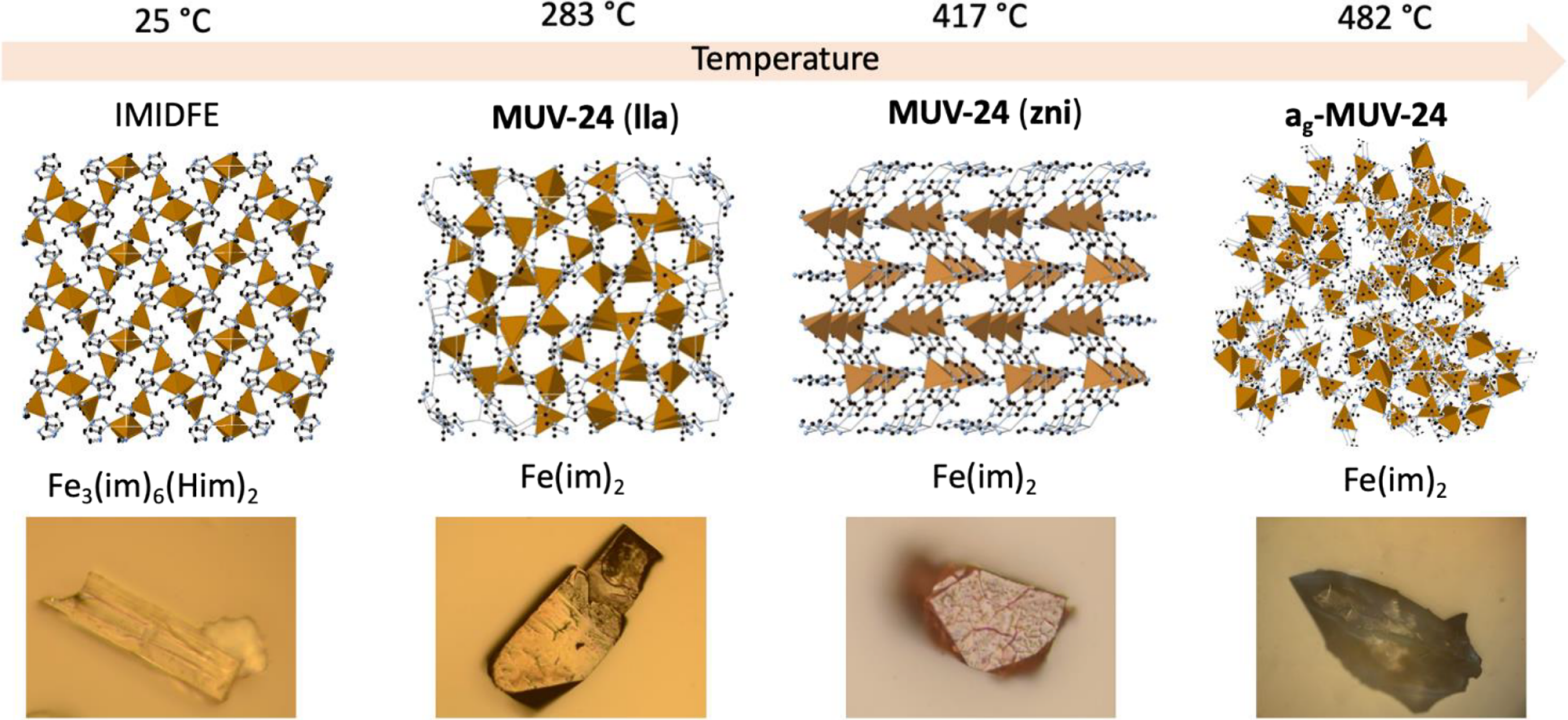
(From the left to right) Crystalline structures of **IMIDFE**, **MUV-24(lla)** and **MUV-24(zni)**, and schematic representation of amorphous **a_g_-MUV-24**, all of them accompanied by their respective microscope images (note that **a_g_-MUV-24** was arbitrary created to illustrate the lack of order). The products formed upon melting and quenching at each temperature represent a clear visual change. Furthermore, in the last material, the vitrification is observed.

The most prominent feature of **MUV-24(lla)** is its novel topology with a net point symbol {4.6.^2^7^3^}_2_{4.6.^4^8}_2_{6.^3^8.^2^9}{6.^4^7.9}, herein named ***lla***. The novelty of the underlying net topology was assessed using TOPOS^35^ and has been registered in the personal topology library. The asymmetric unit includes seven crystallographically different Fe atoms (Figure 2 and Figure S1), all of them acting as 4-connected nodes. Four Fe atoms are linked by im^–^ to form a four-membered ring (Figure S1), which is the smallest ring observed in the structure. In addition, 6-, 7-, 8-, and 9-membered rings are also observed (Figure S1), forming a 3D structure (Figure S2).

Further heating of **MUV-24(lla)** up to 417 °C causes a phase transition, as evidenced by the endothermic peak in the DSC, with no associated mass loss (Figure 1a). Powder X-ray diffraction indicates further structural changes (Figure 1b), showing that the new phase corresponds to the **zni** topology (thus denoted **MUV-24(zni)**), previously reported for Zn^2+^ and Co^2+^ but elusive for Fe^2+^.^36−38^ Significantly, the **zni** topology is an essential intermediate in the melting process of ZIF-4(Zn).^9^ The **zni** topology is considered to be the most thermodynamically stable phase of the Zn-ZIF family,^39^ although other reports identify the **coi** topology as the most stable phase at ambient pressure below 360 °C.^40^ In the case of **MUV-24(zni),** it transforms to the **coi** phase (i.e. to **MUV-24(coi)**) when left at ambient pressure or under vacuum (see Figures S12 and S13), a transformation that is not observed in the Zn^2+^ analogue. Single crystal X-ray diffraction confirms the structure of **MUV-24(coi)**, also reported for Zn^2+^,^37,38^ and Co^2+^,^36^ but not for Fe^2+^.

Upon further heating of **MUV-24(zni)** to 482 °C, another endothermic peak is observed in the DSC, which is also not associated with any mass loss. This peak corresponds with the melting of the material and a viscous material that corresponds with the liquid phase can be observed after the transition. Powder X-ray diffraction of the material after melt-quenching to room temperature shows the absence of Bragg reflections (see Figure 1b), which clearly proves the vitrification of crystalline **MUV-24(zni)** into a noncrystalline phase, denoted **a_g_-MUV-24** (amorphous glass MUV-24), which is nonporous. The melting transformation occurs prior to decomposition of the material at about 530 °C (Figure 1).

These phase transitions were also followed via in situ powder X-ray diffraction (see Figure S4), although we can only clearly observe the **IMIDFE**-to-**MUV-24(lla)** phase transition. This suggests a high sensitivity of the process to the experimental conditions. In fact, upon modification of the DSC conditions (using a hermetic pan), we can prepare **MUV-24(coi)** instead of **MUV-24(zni)**. The importance of minor changes in the thermal process has also been recently reported in the formation of the *qtz*-ZIF-8 phase.^41^

The sequence of structural phase transformations can also be followed through optical images, in which the morphology of the material changes in each of its phases (see Figure 2). Clear differences can be observed between the crystalline and the amorphous phase. In fact, **a_g_-MUV-24** shows evidence of fusion of the microcrystals into a compact monolithic glass.

**Figure 3:**
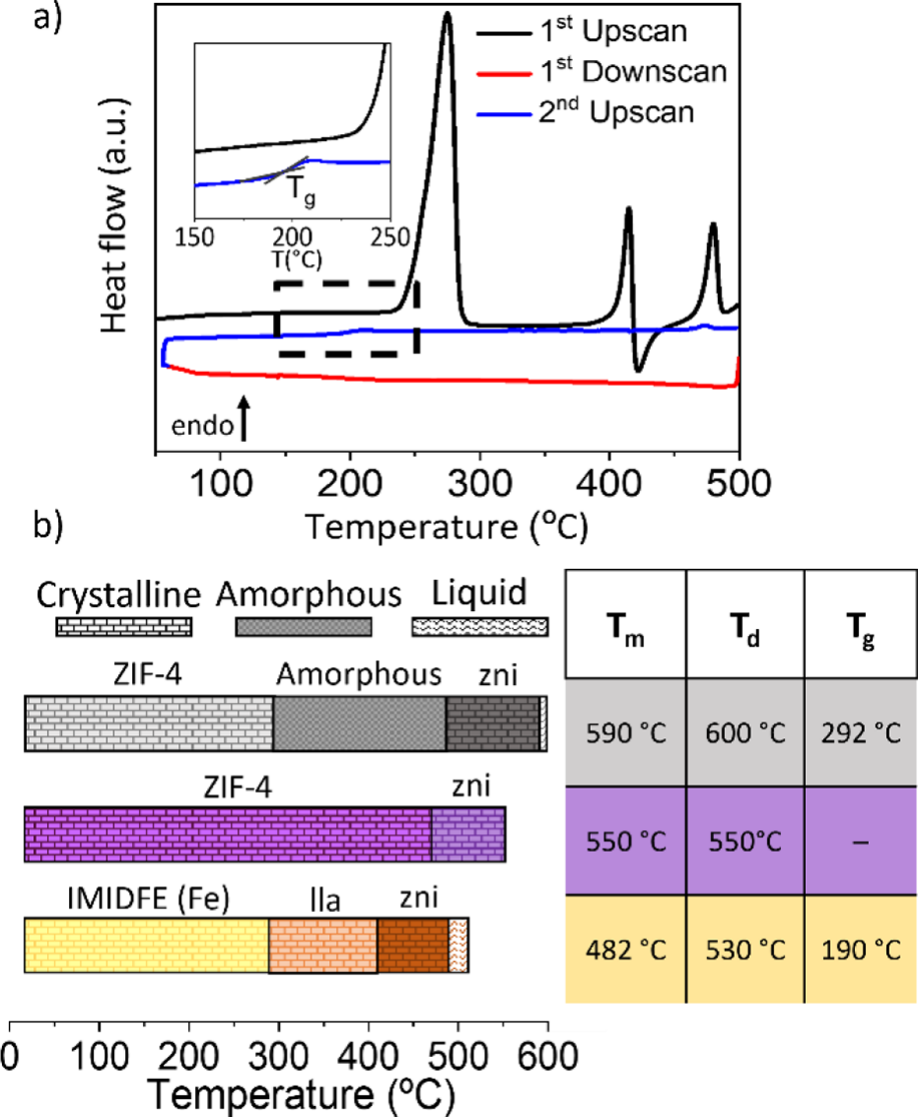
(a) DSC cycles of **IMIDFE** at 10 °C min^–1^; the black line corresponds with the first upscan, the red line corresponds with the first downscan and the blue line corresponds with the second upscan. (b) Schematic representation of the different phase changes undergone by ZIF-4(Zn), ZIF-4(Co) and IMIDFE. ZIF-4(Zn) becomes an amorphous phase, then transforms to the crystalline phase **zni**, which melts and finally decomposes. ZIF-4(Co) is converted to the **zni** crystal phase and decomposes without melting. IMIDFE becomes the **lla** crystalline phase, then transforms into the **zni** phase, which melts and finally decomposes.

Having established the formation of an amorphous phase, we conducted cyclic DSC measurements (two upscans, one downscan) under a N_2_ atmosphere from room temperature to 500 °C at 10 °C min^–1^ (Figure 3) in order to establish the melting point (*T*_m_) of the material, defined as the offset temperature of the calorimetric melting point, and the glass transition temperature (*T*_g_).

The first upscan of the DSC measurement identifies the *T*_m_ of **MUV-24** at 482 °C, which is more than 100 °C lower than the Zn analogue (*T*_m_ = 593 °C)^8^ and 70 °C lower than the Co analogue (*T*_m_ = 550 °C).^23^ Not only is the melting temperature reduced but also the temperature range of the liquid is significantly increased. Thus, the working interval of molten **MUV-24** ranges from 480 to 530 °C (i.e. 50 °C), whereas that of ZIF-4(Zn) is only 7 °C (from 593 to 600 °C)^8^ and nonexistent for ZIF-4(Co) as it decomposes as it melts.^23^

Subsequently, a downscan was performed to obtain the melt-quenched glass and, in a second upscan of **a_g_-MUV-24**, a calorimetric signal associated with the glass transition temperature (*T*_g_) is observed. Similar to what is observed for *T*_m_, *T*_g_ of **a_g_-MUV-24** is significantly lower than *T*_g_ of the a_g_ ZIF-4 (190 °C vs 292 °C)^8^ and still much lower than the lowest reported *T*_g_ (250 °C for Zn(im)_1.87_(6-Cl-5-Fbim)_0.13_).^27^ We believe that the lability of the Fe–im bond is the reason for this clear decrease in *T*_g_.

**Figure 4:**
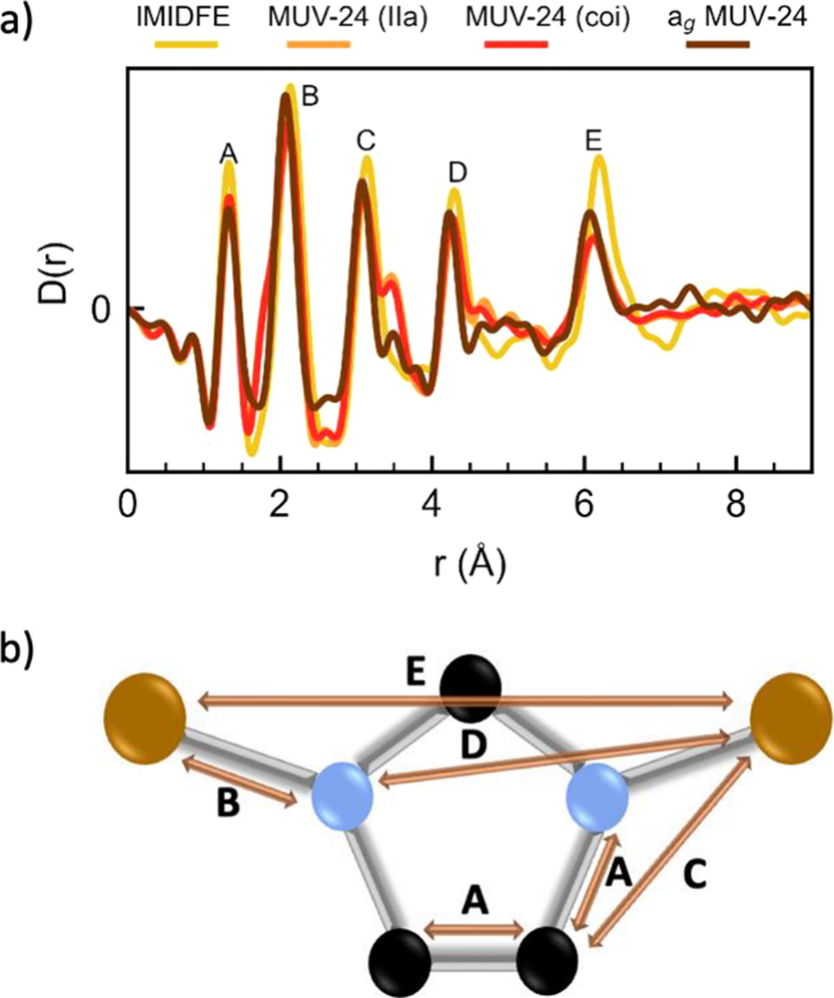
(a) X-ray PDF in the form of *D*(*r*) of **IMIDFE**, **MUV-24(lla)**, **MUV-24(coi)**, and **a_g_-MUV-24**. (b) Structural representation of the short-range order matching bonds and pair distances with the peaks shown in a. Fe, C, and N atoms are shown in brown, black, and blue, respectively.

In order to get further insights into the structural differences and similarities between the different crystalline and amorphous phases of **MUV-24**, X-ray total scattering experiments were performed on **IMIDFE**, **MUV-24(lla)**, **MUV-24(coi)** (as the **zni** phase was not stable at ambient conditions) and **a_g_-MUV-24**. The total scattering structure factors, *S*(*Q*), are given in Figure S6 and show that the Bragg peaks at low-*Q* from the crystalline phases are not present in the **a_g_-MUV-24** data. However, additional Bragg peaks are observed in the **a_g_-MUV-24** data at higher-*Q*. These can be indexed to crystalline impurities of Al and Fe_2_N (Figure S7) and are a small proportion of the scattering signal. The corresponding X-ray pair distribution function (PDF or *D*(*r*)) data are shown in Figures 4 and S8. The similarity in the short-range correlations up to ∼6 Å (corresponding to the distance between neighboring Fe(II) centers) across all the PDFs clearly shows that the tetrahedral coordination of the Fe(II) centers with imidazolate linkers is preserved in **a_g_-MUV-24**.

Furthermore, small differences to the peak at ∼6 Å, reflect differences in the crystal structures (see Figure S9). The broader, more-structured ∼6 Å peak from the **IMIDFE** sample is a consequence of the presence of different Fe centers, in this case, tetrahedral and octahedral. The shorter (tetrahedral) distance remains in the amorphous structure, thus confirming the similar environment in all **MUV-24** phases. There are also two small peaks in the **MUV-24(lla)** and **MUV-24(coi)** PDFs that are not present in the calculated PDFs: a shoulder to peak B at ∼1.8 Å and a peak at ∼3.5 Å next to peak C (Figures 4a and S9). It is difficult to attribute these features to a specific origin. There is no evidence for crystalline impurity in these samples but there could be an amorphous component given that the Bragg peaks from these two phases are much less intense than those from **IMIDFE** (Figure S6). Also, the PDF data from a laboratory-based X-ray diffractometer are not of the highest quality so experimental artifacts, especially at the lowest *r*-values, cannot be ruled out. In addition, the region around *r* ∼ 3.5 Å corresponds to N···N distances within the FeN_4_ tetrahedra and also to distances between (C,N)···(C,N) atom pairs from different imidazole ions that are not linked to a common Fe ion, both of which will be strongly influenced by disorder and distortion within the structures. Finally, we note that the limited *Q*-range of the total scattering data means that the PDF data are of relatively low resolution. This makes a discussion of the detail of the Fe(im)_4_ tetrahedral arrangement difficult, contrary to the recent work based on ^67^Zn NMR measurements.^42^

**Figure 5:**
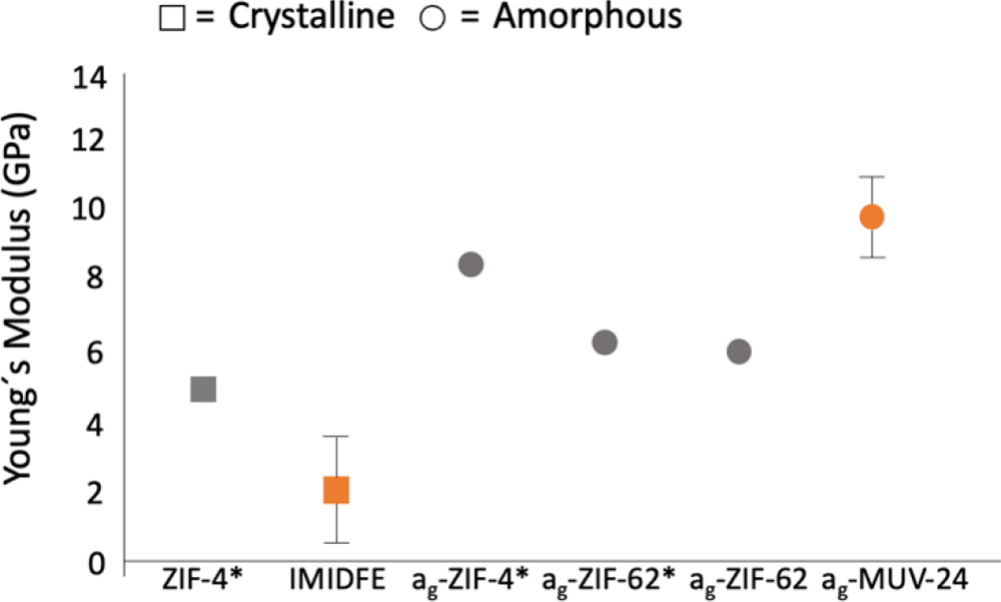
Comparison of Young’s modulus between crystalline (square) and glassy (circle) ZIFs. The gray color represents the ZIFs with Zn centers, while the orange color refers to iron centers. Values marked with an asterisk correspond to previously reported data.^8a^

The formation of glass is normally accompanied by changes in the mechanical properties of the solids, observed as an increase in Young’s modulus (*E*). Different MOF glass specimens have been studied with nanoindentation, typically using a nanoindenter with a diamond tip.^11,43^ Here, we have used, for the first time, an atomic force microscopy (AFM) instrument in PeakForce quantitative nanomechanical property mapping mode (PF-QNM) to measure the Young’s modulus of a monolithic glass. This technique does not destroy or damage the tested material, does not require large bulk glass samples for mechanical testing, nor any kind of treatment of the sample. Moreover, it provides a Young’s modulus mapping of the scanned surface with nanometer resolution, which could be very interesting for identifying the existence of local inhomogeneities. This technique was used to measure both the **IMIDFE** crystalline phase and **a_g_-MUV-24**. We also measured the previously reported a_g_-ZIF-62 in order to verify the validity of the methodology, confirming that the system gives reliable values (Figure 5). In this case, different random faces of **IMIDFE** were measured giving a mean value of *E* = 2.2 ± 1.7 GPa. Upon vitrification to **a_g_-MUV-24**, significant stiffening is observed (*E* = 9.9 ± 1.2 GPa) which represents enhancement of the hardness of this material (Figure 5). This trend is quite similar to that observed for ZIF-4(Zn) and a_g_-ZIF-4(Zn).^8a^

## Conclusions

We have successfully synthesized and characterized the first Fe-ZIF glass, denoted **a_g_-MUV-24**, which is obtained via a three-step structural rearrangement from the known coordination polymer [Fe_3_(im)_6_(Him)_2_], in which octahedral and tetrahedral Fe(II) centers alternate. In the first step, a release of the terminal protonated imidazole ligand occurs, yielding a dense 3D solid with a new topology, denoted **MUV-24(lla)**, with exclusively tetrahedral Fe(II) centers. The second structural transformation causes this solid to rearrange into the well-known zni topology, already reported for Zn(II) and Co(II), but not for Fe(II). Finally, upon further heating **MUV-24(zni)** to 482 °C, the solid melts. This melting temperature for Fe(im)_2_ is significantly below the melting temperature of the Zn analogue, thus potentially improving the applicability of the material. This arises from the incorporation of a more labile metal center such as Fe(II), which is contrary to previous studies of mixing ligands in order to increase the working interval and avoid decomposition. Further tuning of the system with bulkier ligands is currently under exploration and could lead to the formation of very low temperature melts.

## Experimental Section

### Synthesis of IMIDFE

Ferrocene (55.8 mg, 0.3 mmol) and imidazole (40.8 mg, 0.6 mmol) were combined and sealed under vacuum in a layering tube (4 mm diameter). The mixture was heated at 150 °C for 4 days to obtain yellow crystals suitable for X-ray single-crystal diffraction. The product was allowed to cool to room temperature, and the layering tube was then opened. The unreacted precursors were extracted with acetonitrile. **IMIDFE** was isolated as yellow crystals. Phase purity was established by X-ray powder diffraction.

### Synthesis of MUV-24(lla)

Approximately 15 mg of **IMIDFE** was treated with the following thermal process: *T*_initial_ = 40 °C (15 min) → 300 °C → 25 °C. Heating/cooling rate = 10 °C s^–1^.

### Synthesis of MUV-24(zni)

Approximately 15 mg of **IMIDFE** was treated with the following thermal process: *T*_initial_ = 40 °C (15 min) → 430 °C → 25 °C. Heating/cooling rate = 10 °C s^–1^.

### Synthesis of MUV-24(coi)

Approximately 15 mg of **MUV-24(zni)** was sealed under vacuum in a layering tube (4 mm diameter). A progressive transformation of the **zni** phase into the **coi** phase takes place, which is completed in approximately 4 h.

### Synthesis of **a_g_-MUV-24**

Approximately 15 mg of **IMIDFE** was treated with the following thermal process: *T*_initial_ = 40 °C (15 min) → 500 °C → 25 °C. Heating/cooling rate = 10 °C s^–1^.

### Single-Crystal Diffraction

Single crystals of **MUV-24(lla)** and **MUV-24(coi)** were mounted on glass fibers using a viscous hydrocarbon oil to coat the crystals and then transferred directly to the cold nitrogen stream for data collection. X-ray data were collected at 100 K on a DW rotating anode synergy R diffractometer with the (Cu-K_α_) X-ray source (λ = 1.5406 Å). Data were measured using the CrysAlisPro suite of programs. The program CrysAlisPro, Rigaku, was used for unit cell determinations and data reduction. Empirical absorption correction was performed using spherical harmonics, implemented in the SCALE3 ABSPACK scaling algorithm, based upon symmetry-equivalent reflections combined with measurements at different azimuthal angles. The crystal structures were solved and refined against all *F*^2^ values using the SHELXL and Olex2 suite of programs.^44,45^ Atomic displacement parameters of all non-hydrogen atoms were refined anisotropically, except those within a disordered imidazolate ring in each structure, which were refined isotropically. Hydrogen atoms were placed in calculated positions, refined using idealized geometries (riding model), and assigned fixed isotropic atomic displacement parameters. CCDC 2238548–2238549 contain the supplementary crystallographic data for this paper. These data can be obtained free of charge via www.ccdc.cam.ac.uk/conts/retrieving.html (or from the Cambridge Crystallographic Data Centre, 12 Union Road, Cambridge CB21EZ, UK; fax: (+44)1223-336-033; or deposit@ccdc.cam.ac.uk).

### Differential Scanning Calorimetry

Differential scanning calorimetry (DSC) measurements were conducted on a TRIOS DSC 250 instrument. The activated sample (10–15 mg) was loaded into an aluminum crucible (30 μL) with a pierced lid. An empty aluminum crucible was used as a reference. Under N_2_ gas, the sample was heated to a temperature of 40 °C and an isotherm was performed for 15 min to stabilize the sample. Then, the sample was heated to 300, 430, and 500 °C at a rate of 10 °C min^–1^ for **MUV-24(lla)**, **MUV-24(zni)**, and **a_g_-MUV-24**, respectively. Upon reaching the temperature, an isotherm of 10 min was performed to ensure a complete phase change. This was followed by cooling back to 40 °C at 10 °C min^–1^.

### X-ray Total Scattering

X-ray total scattering data were collected at room temperature using the PANalytical Empyrean laboratory diffractometer equipped with an Ag-K_α_ source and focusing mirrors. The data were collected with the sample loaded in a 1 mm diameter quartz glass capillary. For each sample, multiple scans were measured with a total collection time of over 24 h per sample. Similar measurements were made of an empty capillary and the diffractometer background. The resulting X-ray total scattering patterns were processed in the GudrunX program^46^ to produce a normalized PDF optimized such that (for example) the low-*r* portion of *g*(*r*) oscillates around −1. A *Q*_min_ of 0.6 Å^–1^ and *Q*_max_ of 18.5 Å^–1^ were used to obtain the PDF.

### Atomic Force Microscopy

We performed PeakForce Quantitative Nanoscale Mechanical characterization (PF-QNM) in PeakForce Tapping mode, in a Bruker Dimension Icon AFM (Bruker Corporation, CA, USA) to map the topography and the Young’s modulus of different materials. **IMIDFE**, **a_g_-MUV-24**, and ZIF-62 (as reference material) were drop-casted on silicon substrates and imaged, in air under ambient conditions, with RTESPA-150 probes (spring constant 5 N/m, Bruker). The force applied by the tip was fixed to ∼1 nN in all experiments. The automatic analysis of these curves generates maps of mechanical property distribution and topography simultaneously.

## Supporting Information Available

General methods; materials; and crystallographic data (PDF)
