# Supplementary material for: Meltable, Glass-Forming, Iron Zeolitic Imidazolate Frameworks
Source: J Am Chem Soc. 2023 May 9;145(20):11258–64. doi: 10.1021/jacs.3c01455 (PMC10214446; doi:10.1021/jacs.3c01455)
Supplement: Supplementary file 1 — ja3c01455_si_001.pdf [file ja3c01455_si_001.pdf]

A meltable, glass-forming, iron zeolitic imidazolate framework

Luis León-Alcaide,[†] Rasmus S. Christensen,[a] David A. Keen,[‡] José L. Jordá,[⊥] Isaac Brotons-Alcázar,[†] Alicia Forment-Aliaga,[†] Guillermo Mínguez Espallargas*[†]

[†]Instituto de Ciencia Molecular (ICMol), Universidad de Valencia, c/ Catedrático José Beltrán, 2, 46980, Paterna, Spain

[‡] ISIS Facility, Rutherford Appleton Laboratory, Harwell Campus, Didcot, Oxfordshire OX11 0QX, United Kingdom

[a] Center for Integrated Materials Research, Department of Chemistry and iNANO, Aarhus University, Langelandsgade 140, 8000 Aarhus C, Denmark

[⊥] Instituto de Tecnología Química (UPV-CSIC), Universitat Politècnica de València–Consejo Superior de Investigaciones Científicas, Av. de los Naranjos s/n, 46022 Valencia, Spain

Supporting Information

INDEX

S1. Synthesis 3

S2. Crystal structures 4

S.2.1 Crystallographic information 4

S.2.2 Crystal structure of MUV-24(lla) 5

S3. X-ray powder diffraction 7

S3.1. Ex-situ X-ray diffraction 7

S3.2. Variable temperature X-ray diffraction 8

S4. X-ray total scattering 9

S5. Stability study 12

S6. Nuclear Magnetic Resonance (NMR) 18

S6. Differential Scanning Calorimetry (DSC) 19

S6.1 DSC of successive scans 19

S7. Thermogravimetric Analysis (TGA) - Differential Scanning Calorimetry (DSC) – Mass Spectrometry (MS) 23

S8. Scanning Electron Microscopy (SEM) 24

S9. Polarized Light Microscopy 25

S10. Nanomechanical properties characterization by Atomic Force Microscopy (AFM) 28

S11. References 30

S1. Synthesis

All reagents were commercially available and used without further purification.

Synthesis of IMIDFE. Ferrocene (55.8 mg, 0.3 mmol) and imidazole (40.8 mg, 0.6 mmol) were combined and sealed under vacuum in a layering tube (4 mm diameter). The mixture was heated at 150 °C for 4 days to obtain yellow crystals suitable for X-ray single-crystal diffraction. The product was allowed to cool to room temperature, and the layering tube was then opened. The unreacted precursors were extracted with acetonitrile. **IMIDFE** was isolated as yellow crystals. Phase purity was established by X-ray powder diffraction.

The other phases were synthesized using **IMIDFE** as a precursor via different heat treatments, using a differential scanning calorimeter (DSC). Studies were carried out on a DSC250 instrument (TA Instruments) under a constant N_2 flow (50 mL·min^{-1}). In all experiments a punctured capsule was used to avoid overpressure inside the capsule.

Synthesis of MUV-24(lla). Approximately 15 mg of **IMIDFE** were treated with the following thermal process: $T_{initial}$ = 40 °C (15 min) –> 300 °C –> 25 °C. Heating rate = 10 °C·s^{-1}.

Once the process is completed, phase **MUV-24(lla)** is isolated. Phase purity was established by X-ray powder diffraction.

Synthesis of MUV-24(zni). Approximately 15 mg of **IMIDFE** were treated with the following thermal process: $T_{initial}$ = 40 °C (15 min) –> 430 °C –> 25 °C. Heating rate = 10 °C·s^{-1}.

Once the process is completed, phase **MUV-24(zni)** is isolated. Phase purity was established by X-ray powder diffraction.

Synthesis of MUV-24(coi). Approximately 15 mg of **MUV-24(zni)** were sealed under vacuum in a layering tube (4 mm diameter). A progressive transformation of the **zni** phase into the **coi** phase takes place, which is completed in approximately 4 hours. This phase change happens at room temperature and also takes place in air, but it is accompanied by decomposition of the material.

Synthesis of a_g-MUV-24. Approximately 15 mg of **IMIDFE** were treated with the following thermal process: $T_{initial}$ = 40 °C (15 min) –> 500 °C –> 25 °C. Heating rate = 10 °C·s^{-1}.

Once the process is completed, phase **a_g-MUV-24** is isolated. The absence of crystalline phases was established by X-ray powder diffraction.

S2. Crystal structures

S.2.1 Crystallographic information

Single crystals of **MUV-24(lla)** and **MUV-24(coi)** were mounted on glass fibers using a viscous hydrocarbon oil to coat the crystals and then transferred directly to the cold nitrogen stream for data collection. X-ray data were collected at 100 K on a DW rotating anode synergy R diffractometer with (Cu-$K_α$) X-ray source (λ = 1.5406 Å). Data were measured using the CrysAlisPro suite of programs.

Crystal structure determinations and refinements. The program CrysAlisPro, Rigaku, was used for unit cell determinations and data reduction. Empirical absorption correction was performed using spherical harmonics, implemented in the SCALE3 ABSPACK scaling algorithm, based upon symmetry-equivalent reflections combined with measurements at different azimuthal angles. The crystal structures were solved and refined against all F^2 values using the SHELXL and Olex2 suite of programs.[1,2] Atomic displacement parameters of all nonhydrogen atoms were refined anisotropically except those within a disordered imidazolate ring in each structure, which were refined isotropically. Hydrogen atoms were placed in calculated positions, refined using idealized geometries (riding model) and assigned fixed isotropic displacement parameters.

CCDC 2238548 – 2238549 contain the supplementary crystallographic data for this paper. These data can be obtained free of charge via www.ccdc.cam.ac.uk/conts/retrieving.html (or from the Cambridge Crystallographic Data Centre, 12 Union Road, Cambridge CB21EZ, UK; fax: (+44)1223-336-033; or deposit@ccdc.cam.ac.uk).

Table S1. Crystallographic information of compounds **MUV-24(lla)** and **MUV-24(coi)**

Identification code	**MUV-24(lla)**	**MUV-24(coi)**
Empirical formula	$C_{42}H_{42}Fe_7N_{28}$	$C_{24}H_{24}Fe_4N_{16}$
Formula weight	1329.98	759.99
Temperature/K	120(1)	120(1)
Crystal system	monoclinic	tetragonal
Space group	$P2_1/c$	$I4_1$
a/Å	12.3584(6)	22.5943(4)
b/Å	23.6435(16)	22.5943(4)
c/Å	19.5565(9)	13.2966(4)
$α$/°	90	90
$β$/°	93.237(4)	90
$γ$/°	90	90
Volume/Å^3	5705.2(5)	6787.9(3)
Z	4	8
$ρ_{calc}$/g·cm^{-3}	1.548	1.487
μ/mm^{-1}	14.348	13.782
F(000)	2688.0	3072.0
Crystal size/mm^3	0.02 × 0.02 × 0.02	0.02 × 0.02 × 0.02

Radiation	Cu Kα (λ = 1.54184)	Cu Kα (λ = 1.54184)
2Θ range for data collection/°	5.87 to 133.624	5.532 to 133.108
Index ranges	-14 ≤ h ≤ 13, -28 ≤ k ≤ 28, -23 ≤ l ≤ 23	-26 ≤ h ≤ 26, -26 ≤ k ≤ 17, -15 ≤ l ≤ 15
Reflections collected	39268	21584
Independent reflections	10051 [R_{int} = 0.1714, R_{sigma} = 0.1530]	5961 [R_{int} = 0.0611, R_{sigma} = 0.0547]
Data/restraints/parameters	10051/0/692	5961/1/415
Goodness-of-fit on F^2	1.025	1.055
Final R indexes [I>=2σ (I)]	R_1 = 0.0768, wR_2 = 0.1638	R_1 = 0.0550, wR_2 = 0.1435
Final R indexes [all data]	R_1 = 0.1662, wR_2 = 0.2030	R_1 = 0.0735, wR_2 = 0.1538
Largest diff. peak/hole / e Å^{-3}	0.54/-0.77	0.82/-0.41

S.2.2 Crystal structure of MUV-24(lla)

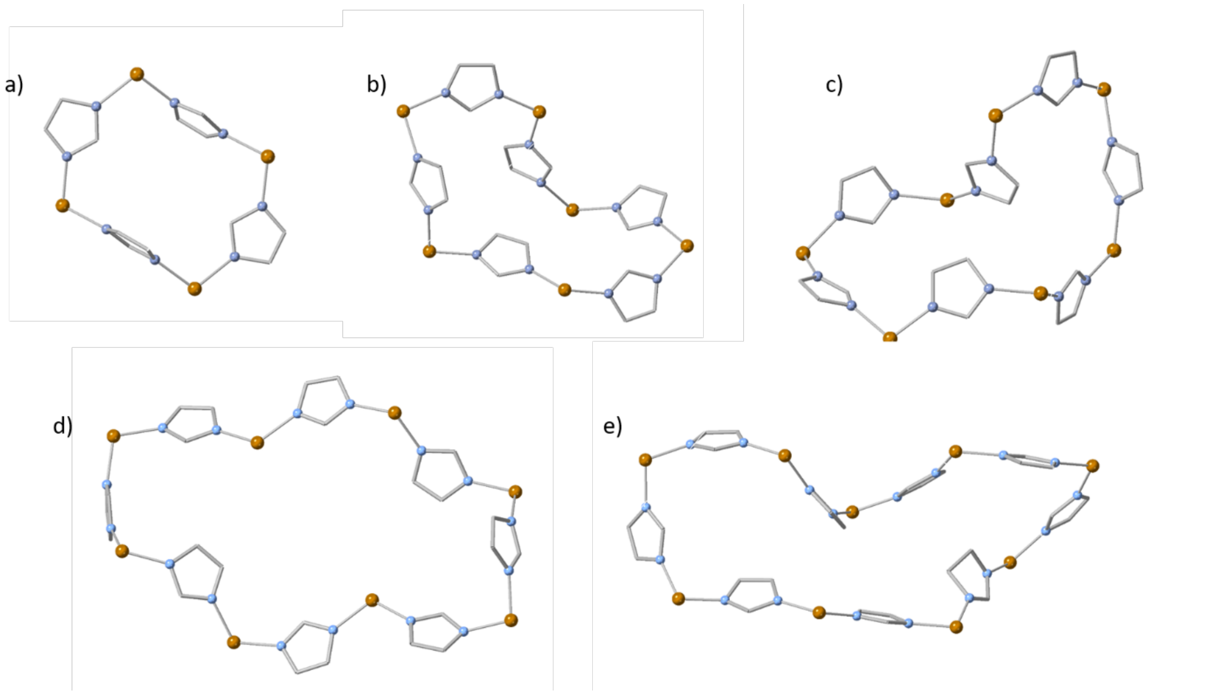

Figure S1: Images of the different rings found in **MUV-24(lla)**. a) 4-member ring (i.e. formed by four iron centers); b) 6-member ring (i.e. formed by six iron centers); c) 7-member ring (i.e. formed by seven iron centers); d) 8-member ring (i.e. formed by eight iron centers); (e) 9-member ring (i.e. formed by nine iron centers). Iron and nitrogen atoms are represented by brown and blue spheres, respectively. Hydrogens have been omitted for clarity.

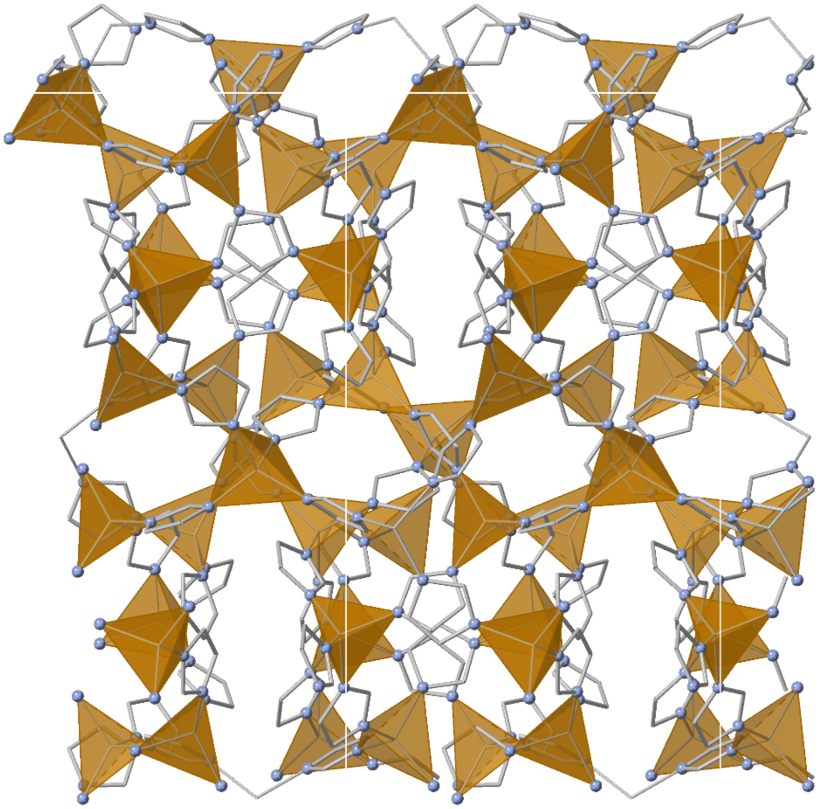

Figure S2. 3D crystalline structure of **MUV-24(lla)** along the *c*-axis. Iron atoms are represented by brown tetrahedrons. The blue balls represent nitrogen atoms. Hydrogen atoms have been omitted for clarity.

S3. X-ray powder diffraction

S3.1. Ex-situ X-ray diffraction

All materials were characterized by X-ray powder diffraction. The samples were lightly ground in an agate mortar and pestle and used to fill 0.5 mm borosilicate capillaries that were mounted and aligned on an Empyrean PANalytical powder diffractometer, using Cu K_α radiation (λ = 1.54056 Å). Repeated measurements were collected at room temperature in the range 2θ = 5–25° and merged into a single diffractogram.

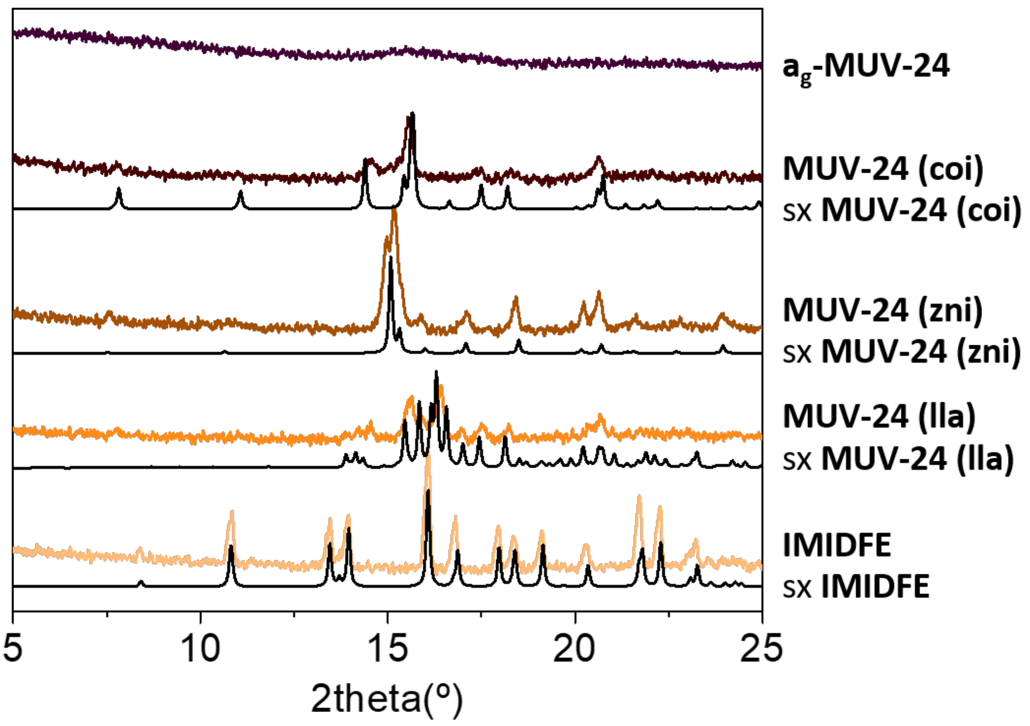

Figure S3. X-ray powder diffraction of **a_g-MUV-24**, **MUV-24(coi)**, **MUV-24(zni), MUV-24(lla)** and **IMIDFE**. The color scale represents the different polymorphs, while the black lines represent the theoretical powder patterns of each phase.

S3.2. Variable temperature X-ray diffraction

Temperature dependent *in situ* X-ray powder diffraction (PXRD) data was collected using an Anton Parr XRK-900 chamber attached to a PANalytical Empyrean diffractometer, to follow the evolution of the sample. Measurements were performed using Cu K_{α} radiation.

The sample was heated under a dry air atmosphere from 25 °C to 400 °C with a heating rate of 10 °C·min^{-1}. At the selected temperatures (see Figure S4) the ramp was stopped, and the temperature was kept constant during the measurement.

It is relevant to note that the transition temperatures observed by PXRD do not coincide with those observed by the DSC. It can be explained by the fact that both measurements are performed in different conditions. In the PXRD experiments the temperature was allowed to stabilize and kept constant during the measurement of each temperature point, keeping the sample in a static condition. However, in the DSC experiment the temperature was continuously increasing during the measurements, causing that the kinetics of the process could impact on the results introducing a small delay in the transitions observed.

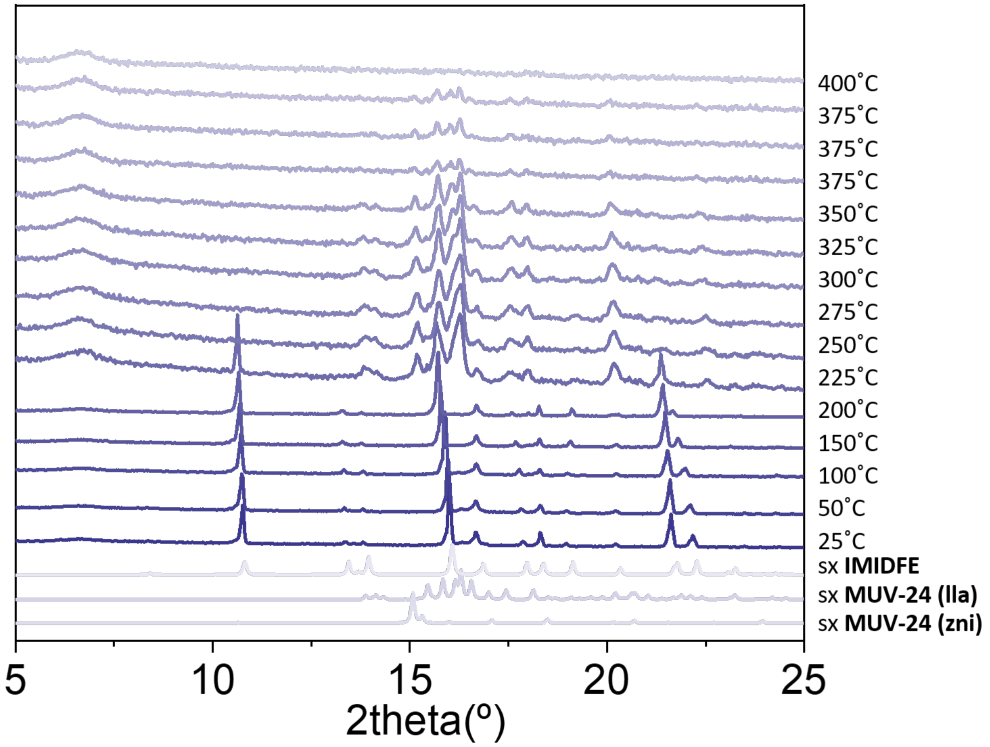

Figure S4. Variable temperature X-ray diffraction of **IMIDFE**. The phase change from **IMIDFE** to **MUV-24(lla)** begins to be observed in the step between 200–225 °C, ending the phase change at 300 °C. The **lla** phase remains stable up to 375 °C, when the crystalline structure is lost. The temperatures do not coincide with what is observed in the DSC, probably due to the fact that the control of the conditions is much less strict.

S4. X-ray total scattering

X-ray total scattering data were collected at room temperature using the PANalytical Empyrean laboratory diffractometer equipped with an Ag-K_α source and focusing mirrors. The data were collected with the sample loaded in 1 mm diameter quartz glass capillaries. For each sample multiple scans were measured with a total collection time of over 24 hours per sample. Similar measurements were made of an empty capillary and the diffractometer background. The resulting X-ray total scattering patterns were processed in the GudrunX program[3] to produce a normalised PDF optimised such that (for example) the low-r portion of g(r) oscillates around –1 (see Figure S5).

A Q_{min} of 0.6 $Å^{-1}$ and Q_{max} of 18.5 $Å^{-1}$ were used to obtain the PDF.

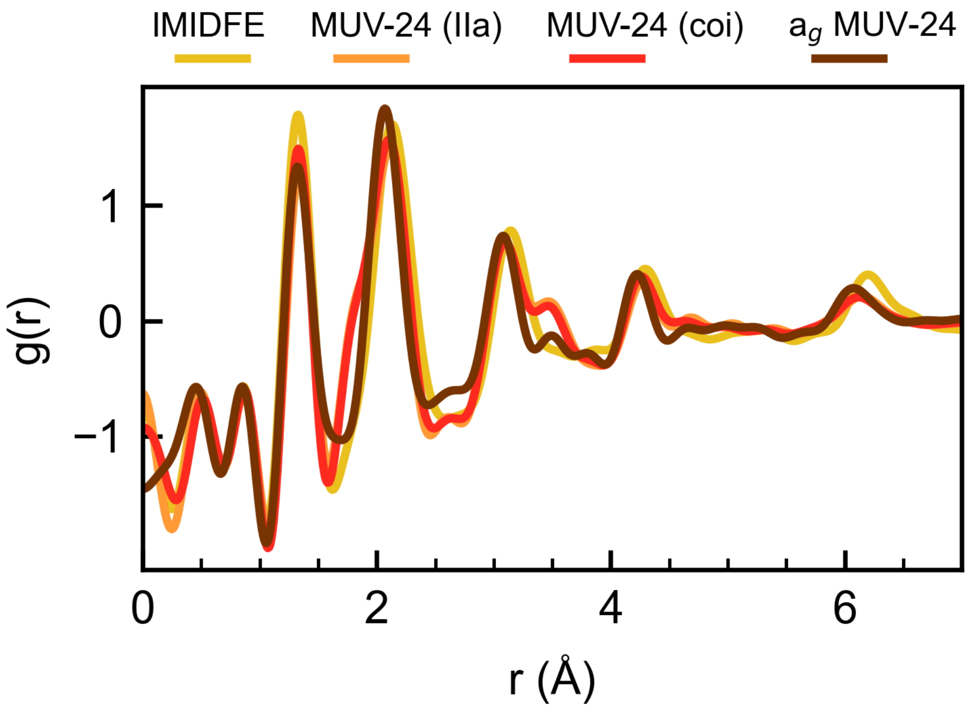

Figure S5: XPDF in the form of g(r) of **IMIDFE**, **MUV-24(IIa)**, **MUV-24(coi)** and **a_g-MUV-24**.

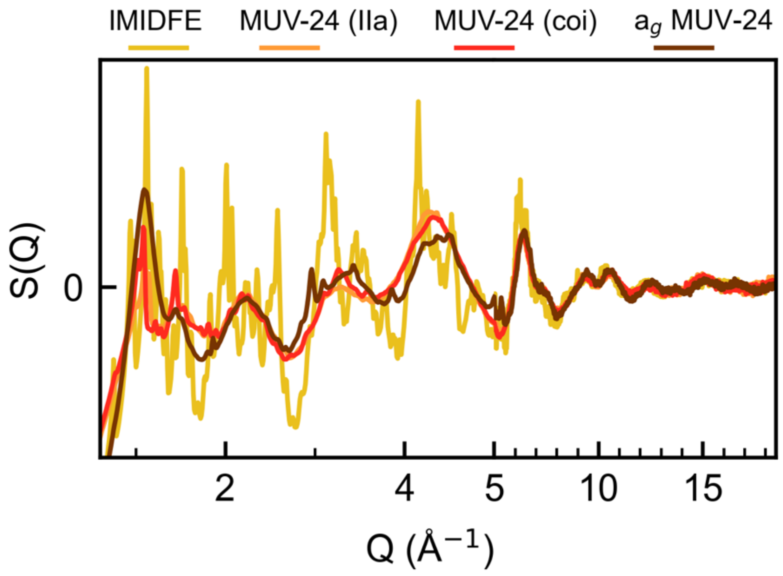

Figure S6: Total scattering structure factors, S(Q), of **IMIDFE**, **MUV-24(lla)**, **MUV-24(coi)** and **a_g-MUV-24**.

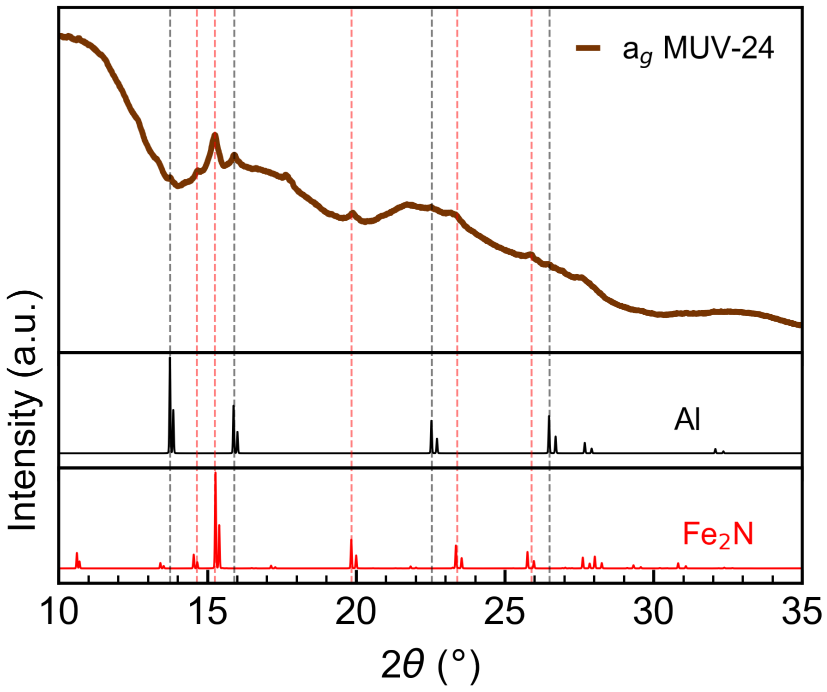

Figure S7: Identification of the crystalline impurities found in **a_g-MUV-24** as Al (from the aluminium DSC pan used in the synthesis, which needs to be broken for recovering the material) and Fe_2N (from partial decomposition of the material).

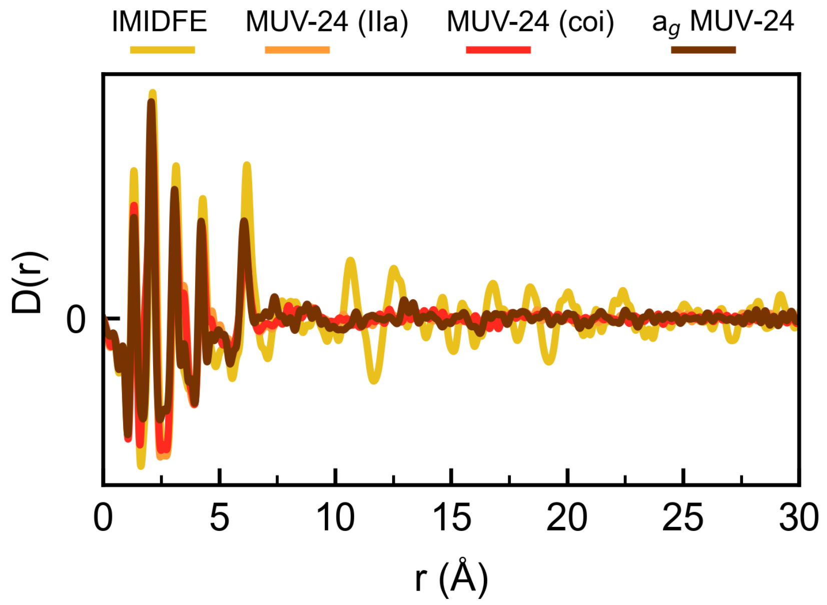

Figure S8: X-ray PDF in the form of D(r) of **IMIDFE**, **MUV-24(lla)**, **MUV-24(coi)** and **a_g-MUV-24**.

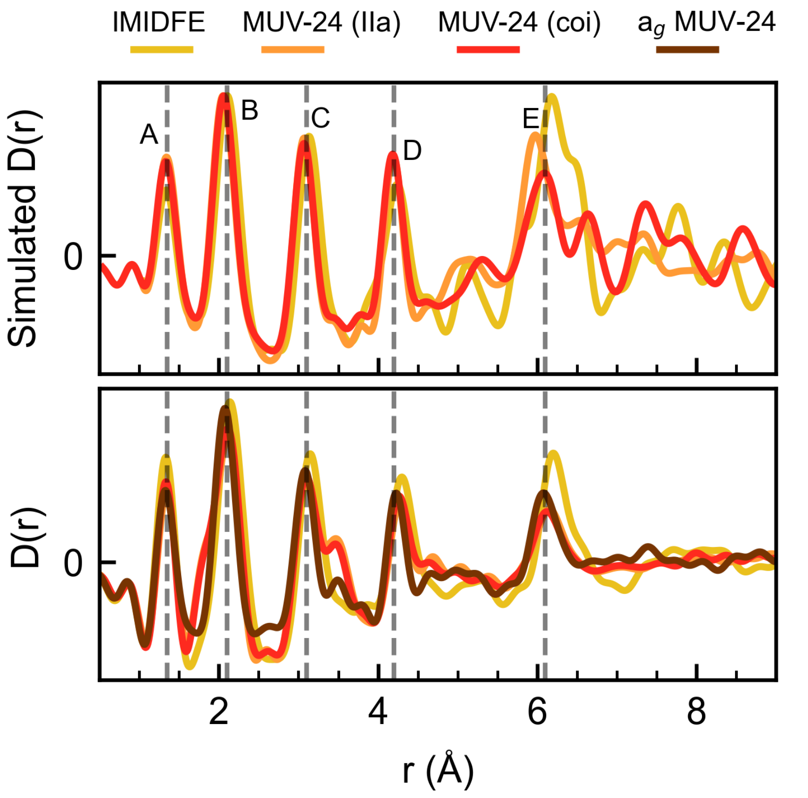

Figure S9: Simulated (top) and experimental (bottom) X-ray PDF in the form of D(r) of **IMIDFE**, **MUV-24(lla)**, **MUV-24(coi)** and **a_g-MUV-24**, highlighting the differences in the peak at 6 Å.

S5. Stability study

Stability studies of each phase were carried out by X-ray diffraction. The stability in all crystalline materials is observed by the loss of crystallinity (i.e. a reduction in the intensity of the peaks), in addition to the recrystallization of the imidazole ligand (except in the case of **a_g-MUV-24**, where only recrystallization of the ligand is observed).

MUV-24(zni) is not stable at room temperature, regardless of whether the atmosphere is air or nitrogen. A study is carried out with powder diffraction both in vacuum and in air. The rest of the materials can be kept in an inert atmosphere without undergoing any change.

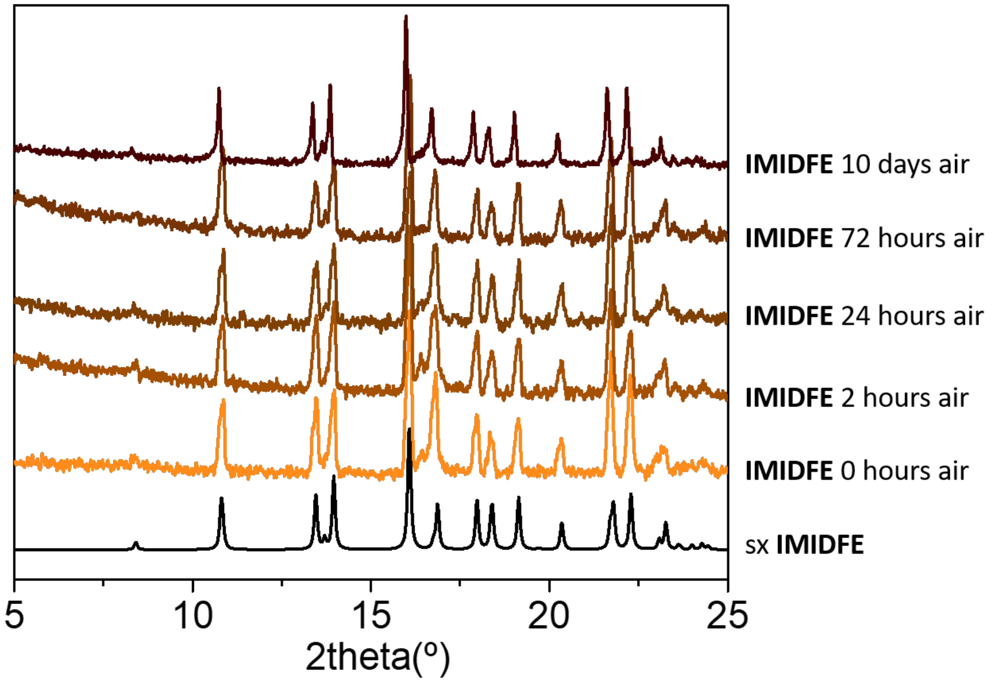

Figure S10. X-ray powder diffraction study of the stability in air of **IMIDFE.** The material was measured at 0 h, 2 h, 24 h, 72 h and 10 days of exposure to air without losing the crystalline structure. However, the colour of the material is observed to darken over time.

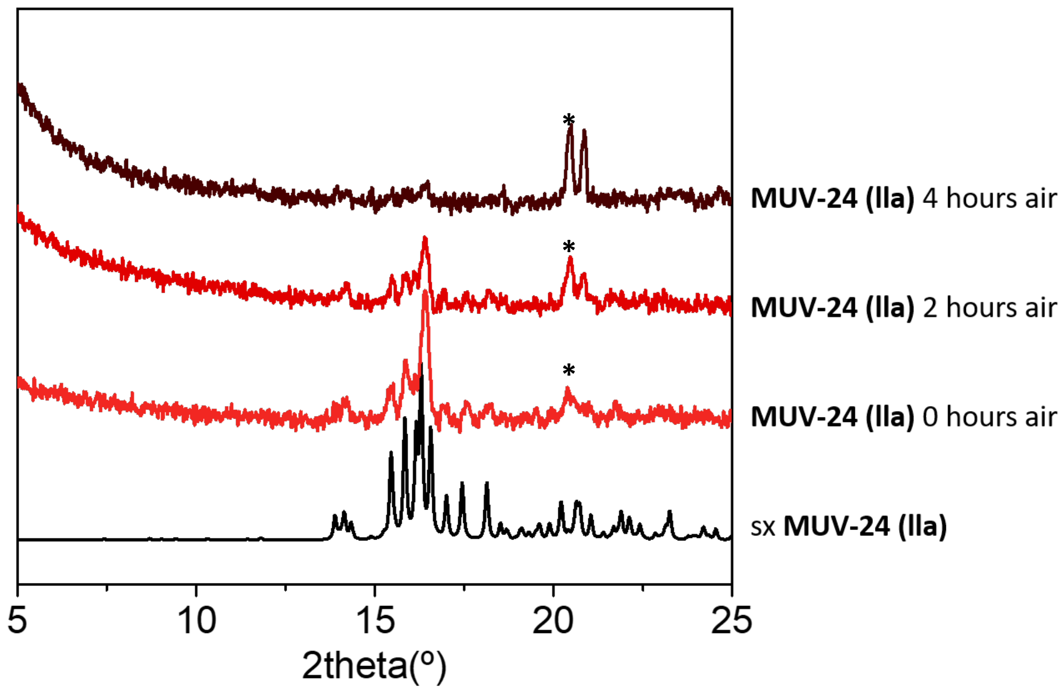

Figure S11. X-ray powder diffraction study of the stability in air of **MUV-24(IIa).** The material was measured at 0 h, 2 h and 4 h of exposure to air with a complete loss of the peaks that correspond with the crystalline structure of **MUV-24(IIa)** and the appearance of new peaks that correspond to the recrystallization of the imidazole ligand (marked with an asterisk).

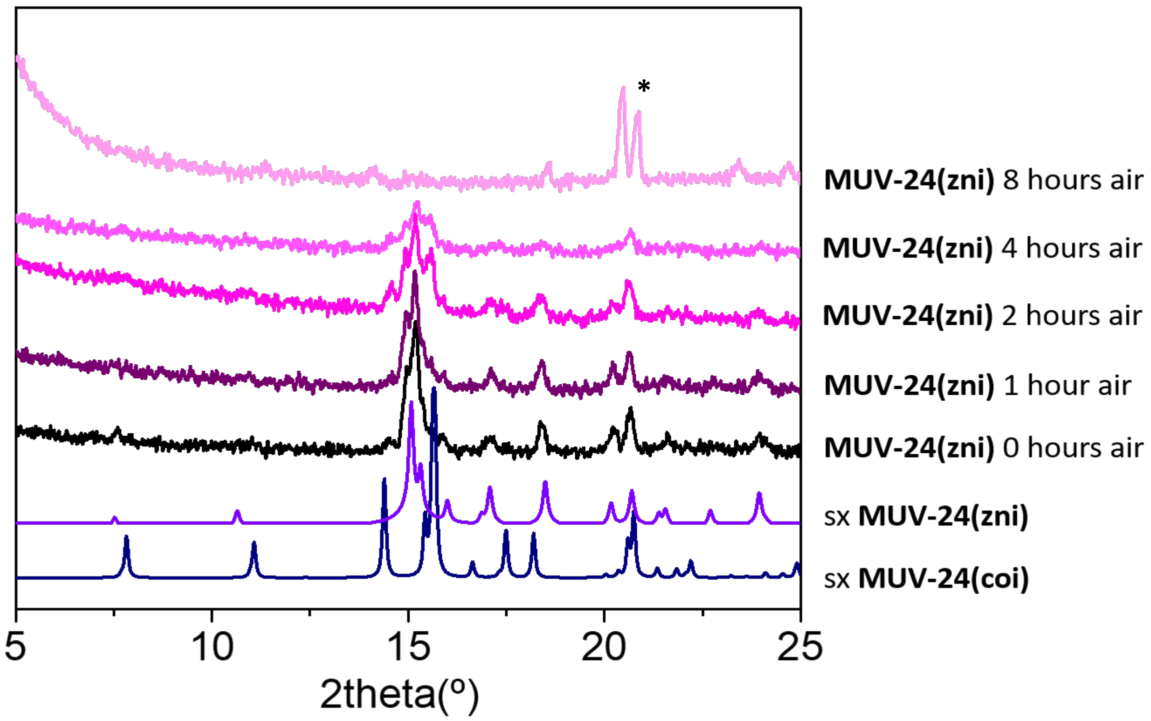

Figure S12. X-ray powder diffraction study of the stability in air of **MUV-24(zni).** The material was measured at 0 h, 1 h, 2 h, 4 h and 8 h of exposure to air. The partial conversion of **MUV-24(zni)** into **MUV-4(coi)** is observed over time. Contrary to the evolution of **MUV-24(zni)** under vacuum (with a complete conversion to **MUV-24(coi)**, see Figure S13 below), complete conversion of the **zni** to **coi** phase is never achieved in air due to degradation of both phases. After 8 h a complete loss of the peaks that correspond wither to the crystalline structure of **MUV-24(zni)** or **MUV-24(coi)** is observed, with the appearance of new peaks that correspond to the recrystallization of the imidazole ligand (marked with an asterisk).

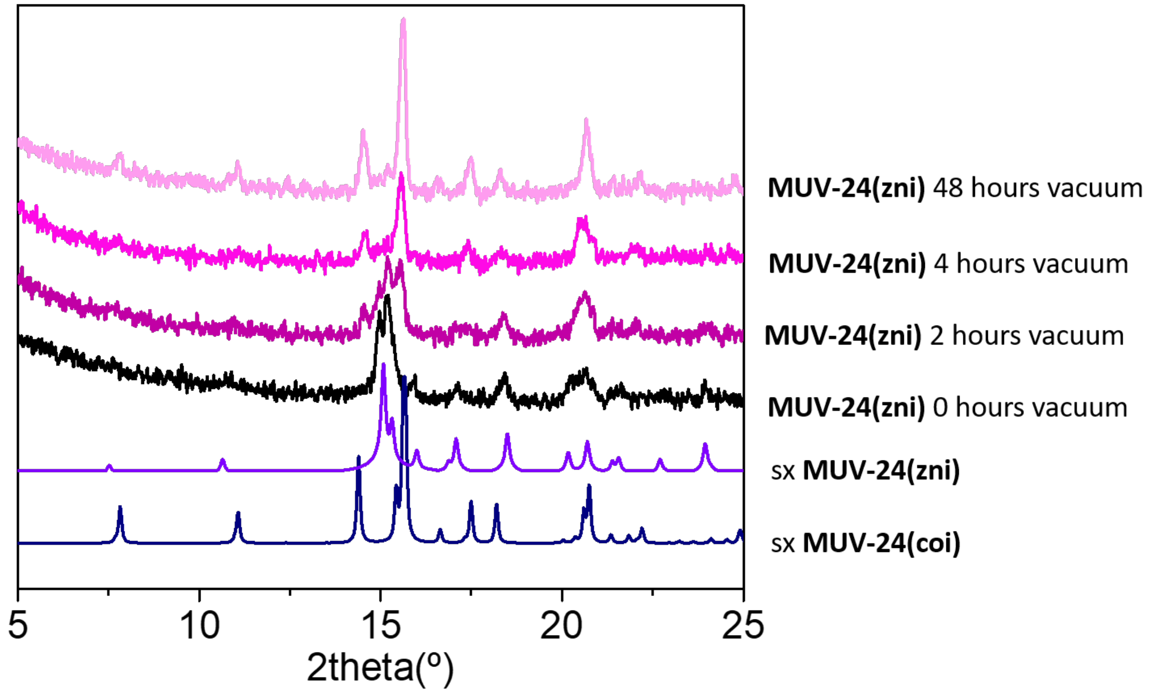

Figure S13. X-ray powder diffraction study of the stability in vacuum of **MUV-24(zni).** The material was measured at 0 h, 2 h, 4 h, 48 h of exposure to vacuum. The partial conversion of **MUV-24(zni)** into **MUV-4(coi)** is observed over time. A complete conversion of the **zni** to **coi** phase is achieved after 4 h. The material is stable under these conditions and does not undergo any further transformation.

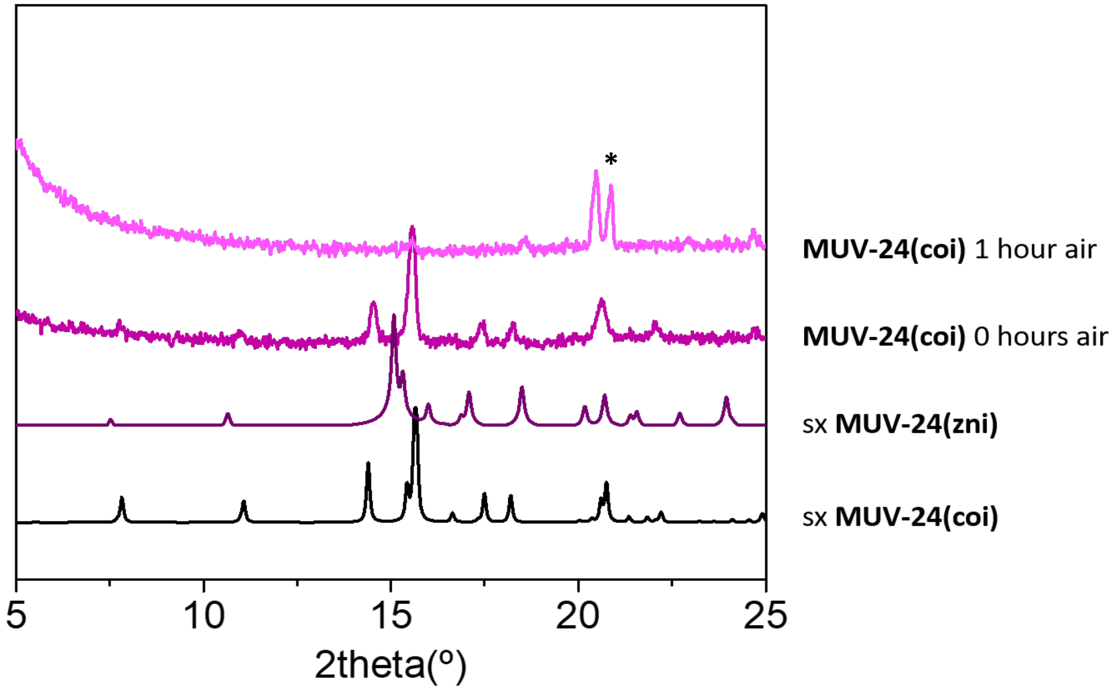

Figure S14. X-ray powder diffraction study of the stability in air of **MUV-24(coi).** The material was measured at 0 h and 1 h of exposure to air with a complete loss of the peaks that correspond with the crystalline structure of **MUV-24(lla)** and the appearance of new peaks that correspond to the recrystallization of the imidazole ligand (marked with an asterisk).

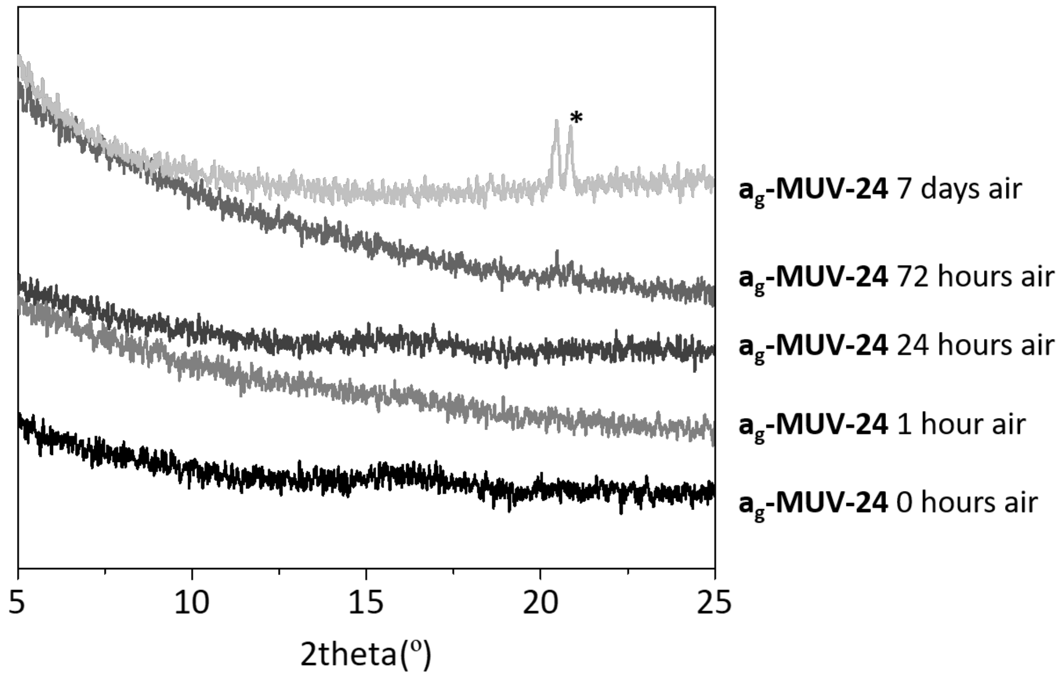

Figure S15. X-ray powder diffraction study of the stability in air of **a_g-MUV-24.** The material was measured at 0 h, 1 h, 24 h and 72 h of exposure to air. As this material presents an amorphous structure, we could only analyze the recrystallization of the ligand with time (marked with an asterisk).

S6. Nuclear Magnetic Resonance (NMR)

NMR spectra were recorded on Bruker DRX-500 spectrometer. The ligand ratio was confirmed by ^{1}H NMR spectroscopy (upon digestion in D_2O and deuterated trifluoroacetic acid).

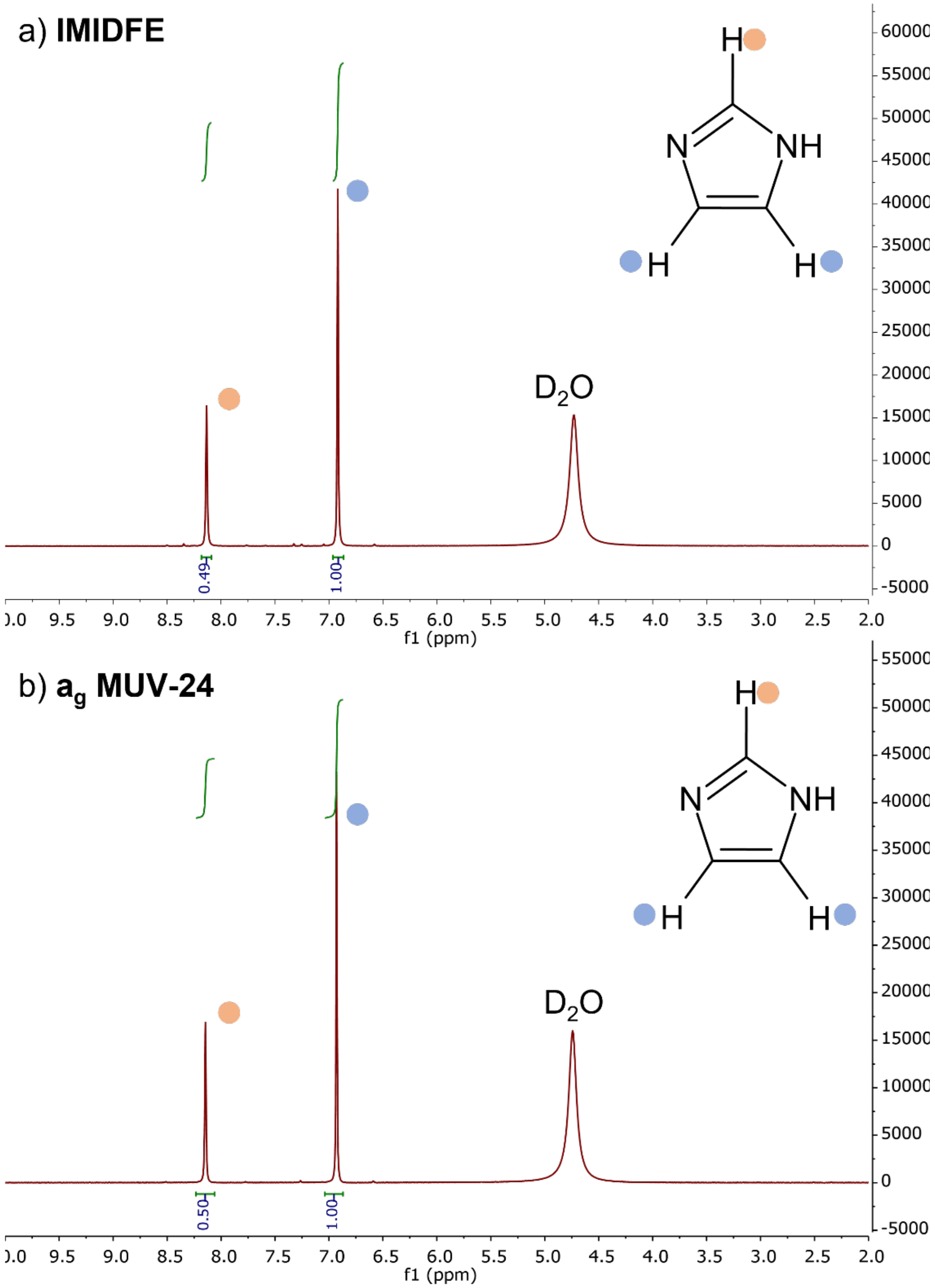

Figure S16. ^{1}H NMR spectra of digested **IMIDFE** and **a_g MUV-24**, where it can be clearly identified the H atoms from imidazole ligand. Coloured circles help to localize the corresponding chemical shifts of the protons.

S6. Differential Scanning Calorimetry (DSC)

Differential scanning calorimetry (DSC) measurements were conducted on a TRIOS DSC 250 instrument. 10-15 mg of activated sample was loaded into an aluminium crucible (30 µL), with a pierced lid.

S6.1 DSC of successive scans

An empty aluminium crucible was used as a reference. Under N_2 gas, the sample was heated to an initial temperature of 40 °C and an isotherm is performed for 15 min to stabilize the sample. Then the sample was heated to 300 °C, 430 °C and 500 °C at a rate of 10 °C·min^{-1} for **MUV-24(lla)**, **MUV-24(zni)** and **a_g-MUV-24**, respectively. Upon reaching the temperature, an isotherm of 10 min is performed to ensure a complete phase change. This was followed by cooling back to 40 °C at 10 °C·min^{-1}. It is important to mention that the results are very dependent on the conditions of the DSC. Thus, upon variation of the equipment it is necessary to modify parameters, such as the nitrogen flow, amount of sample or size of the hole in the pan to obtain the different phase changes.

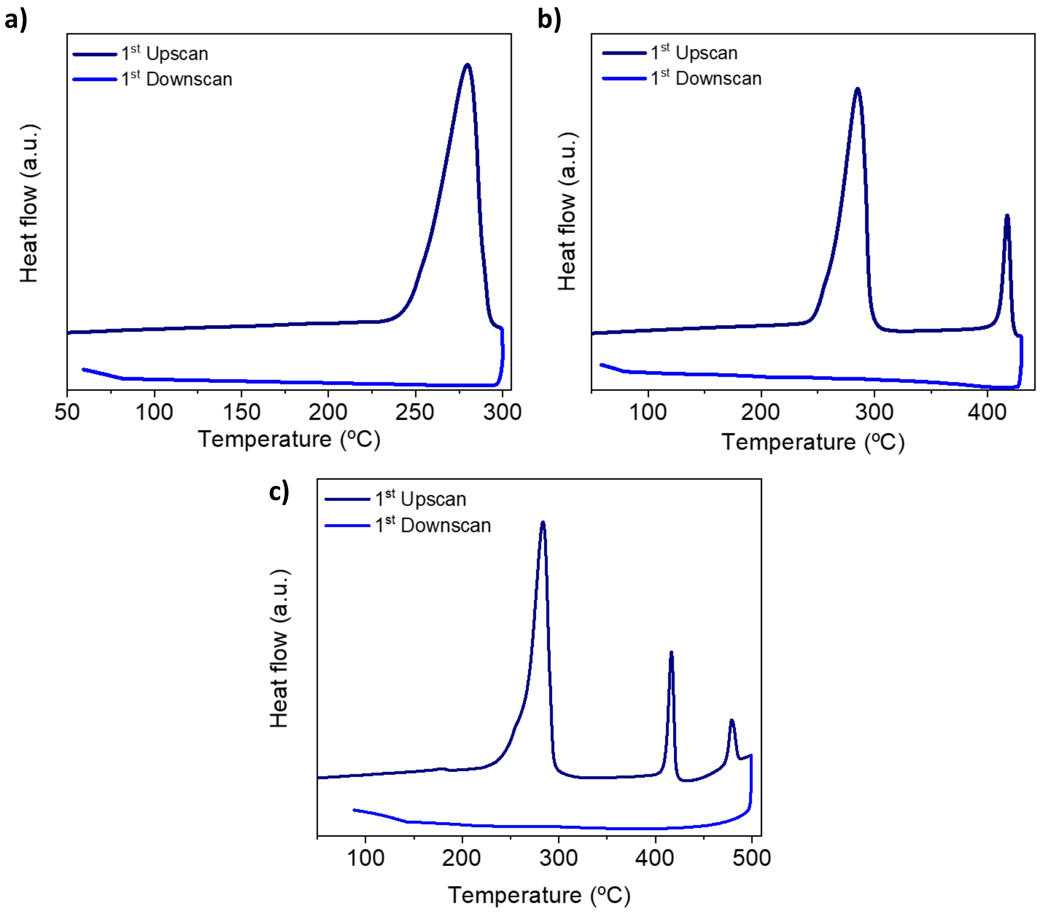

Figure S17. DSC measurements for the preparation of (a) **MUV-24(lla)**, (b) **MUV-24(zni)** and (c) **a_g-MUV-24**. The first scan (dark blue) contains endotherm peaks associated with each phase transition. Subsequent second scan (light blue) was performed upon cooling to room temperature at 10°C·min^{-1}.

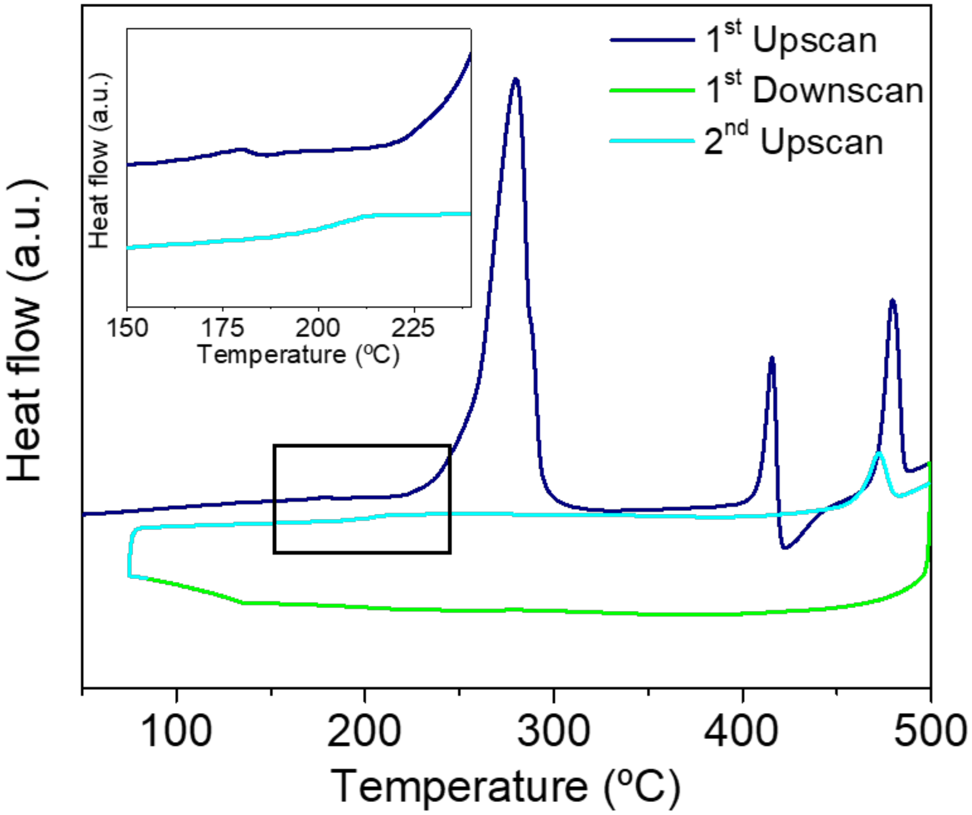

Figure S18. Successive DSC upscans and downscan on **IMIDFE** to 500 °C at a rate of 10 °C·min^{-1} in the absence of an isotherm at 500 °C. In this case the isotherm is not performed to observe how a new melting appears in the second upscan at 480 °C due to either partial recrystallization or incomplete melting in the first upscan. The first upscan (dark blue) contains three endotherm peaks: the first one corresponds to the discoordination of neutral imidazole ligand and the formation of **MUV-24(lla)** phase; the second endothermic peak corresponds to the formation of the **MUV-24(zni)** phase; the third endothermic peak corresponds to the melting. Subsequent downscan (green line) is performed followed by a second upscan (light blue) in which the first two endothermic peaks are not observed (with a glass transition at 190 °C, shown in the zoom area), but a melting process is observed at 480 °C with much lower intensity.

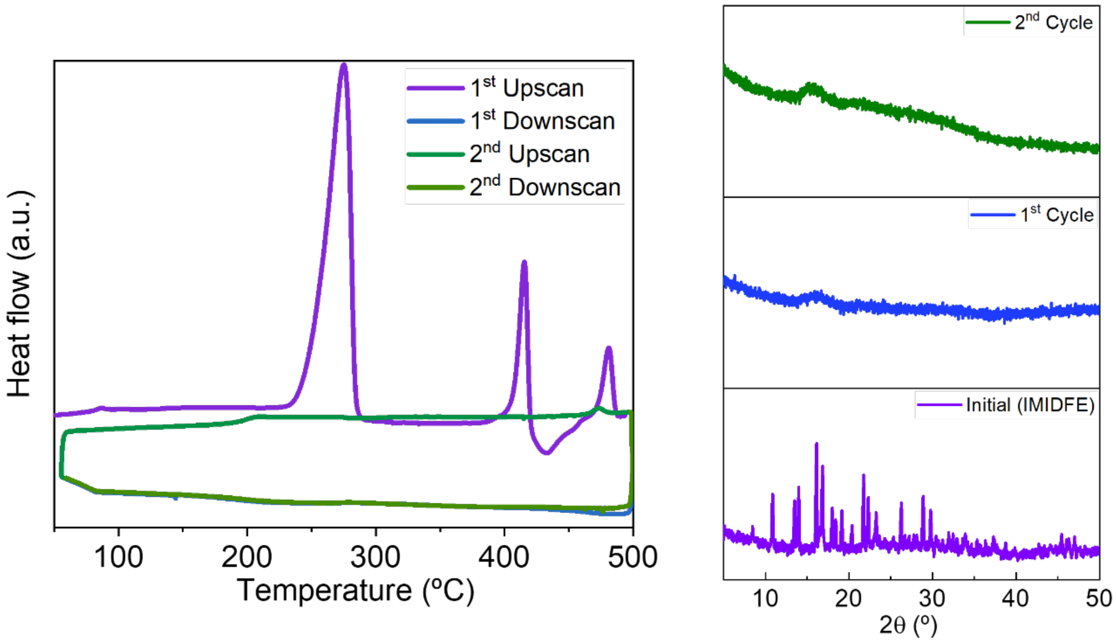

Figure S19. (left) DSC cycles of **IMIDFE** at 10 °Cmin^{-1}; the purple line corresponds with the first upscan, the blue line corresponds with the first downscan, the green line corresponds with the second upscan and the dark green line corresponds to the second downscan. (right) X-ray powder patterns of the different materials: initial compound (in purple), after the first DSC cycle (in blue) and after the second DSC cycle (in green).

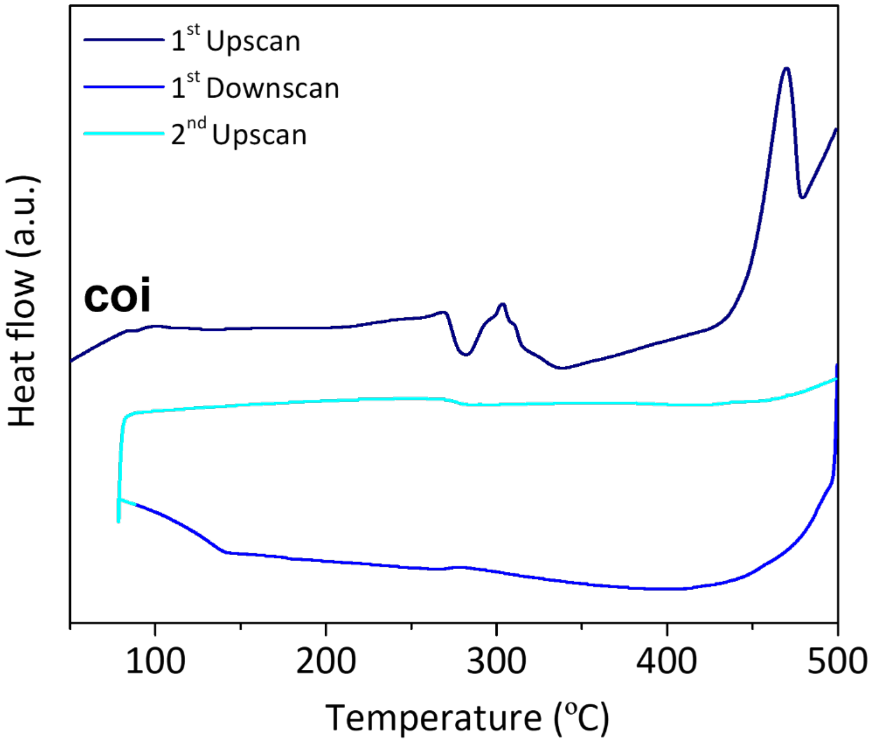

Figure S20. DSC cycles of **MUV-24-coi** at 10 °Cmin^{-1}.

S7. Thermogravimetric Analysis (TGA) - Differential Scanning Calorimetry (DSC) – Mass Spectrometry (MS)

TGA-DSC-MS analysis was performed in simultaneous TG-DSC equipment coupled to a mass spectrometer, using a NETZSCH brand TGA/STS 449 F5 Jupiter coupled to a NETZSCH brand Aeolos QMS 403 Quadro model quadrupole mass spectrometer. The same conditions were used as in the DSC with the subsequent characterization of the volatiles released by mass spectrometry.

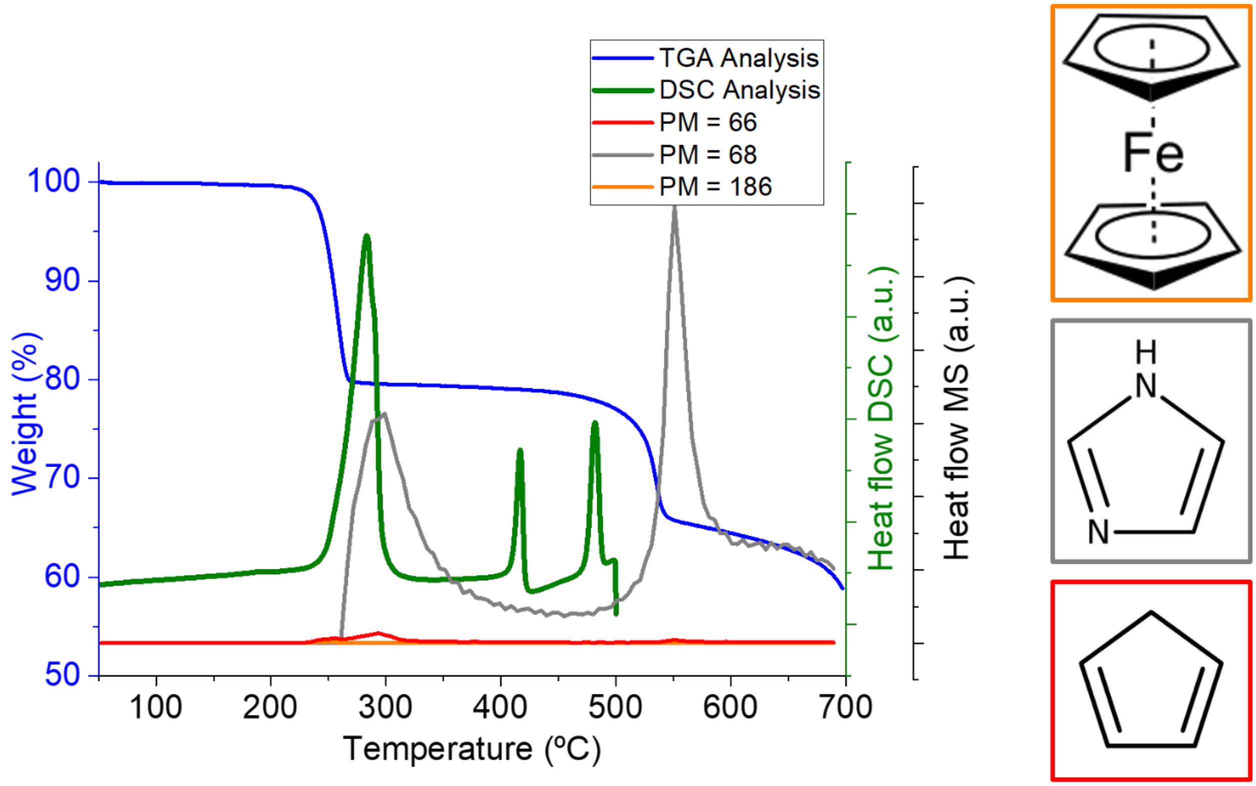

Figure S21. TG-DSC-MS study of **IMIDFE**. The blue line corresponds with the TGA analysis. The green line corresponds with the DSC analysis. The orange, grey and red lines correspond with the mass of the ferrocene, imidazole and cyclopentadienyl, respectively. It is observed how only mass losses associated with imidazole appear, discarding the presence of unreacted ferrocene. The final decomposition of the material, at 530 °C, is also associated with the loss of imidazole.

S8. Scanning Electron Microscopy (SEM)

Scanning Electronic Micrographs image were recorded in a SCIOS 2 FIB-SEM. The synthesis conditions of the materials were the previously established using the DSC equipment.

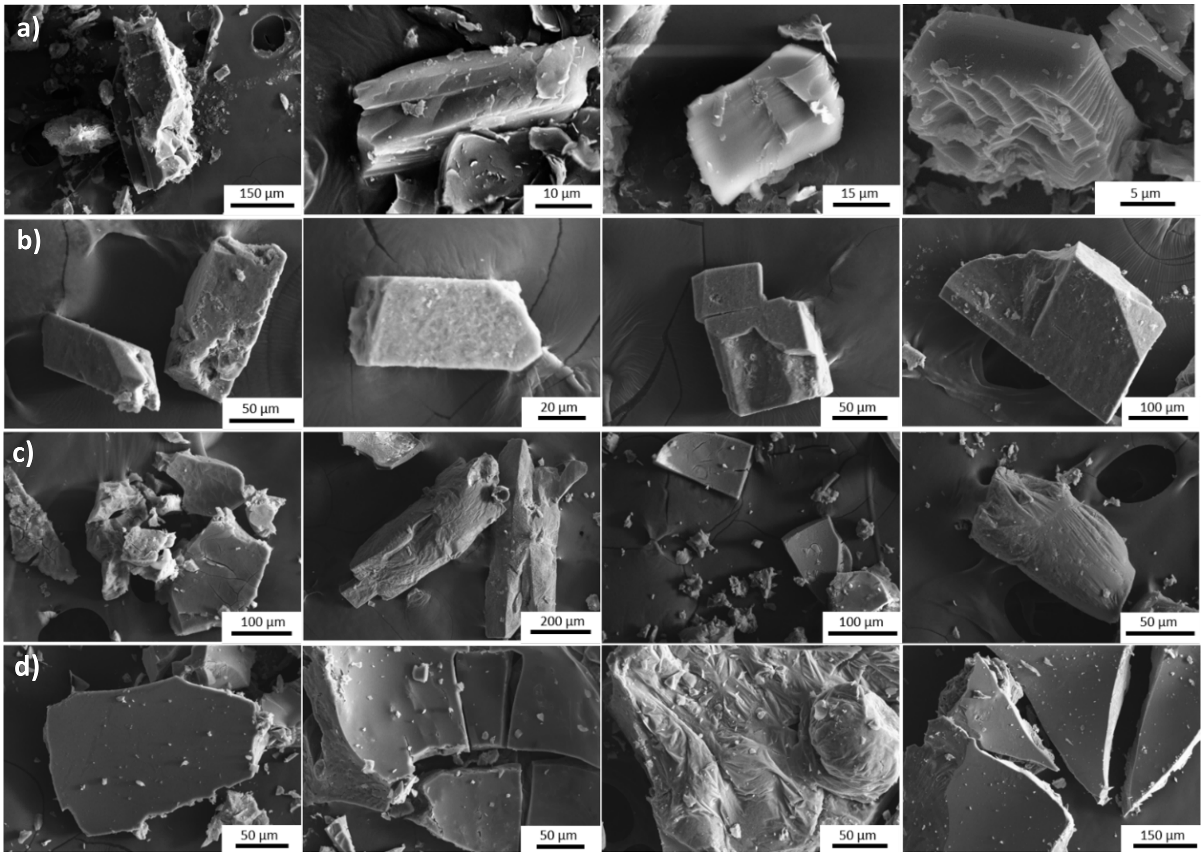

Figure S22. SEM image of the different polymorphs: (a) **IMIDFE**; (b) **MUV-24(lla)**; (c) **MUV-24(coi)**; (d) **a_g-MUV-24**. It can be seen how structures with a more defined morphology are observed in crystalline materials, while **a_g-MUV-24** looks like a compact monolith.

S9. Polarized Light Microscopy

Optical images were obtained with a Nikon Eclipse LV-100 Optical microscope. Pictures of one grain **IMIDFE**, **MUV-24(lla)**, **MUV-24(zni)**, **MUV-24(coi)** and **a_g-MUV-24** are reported.

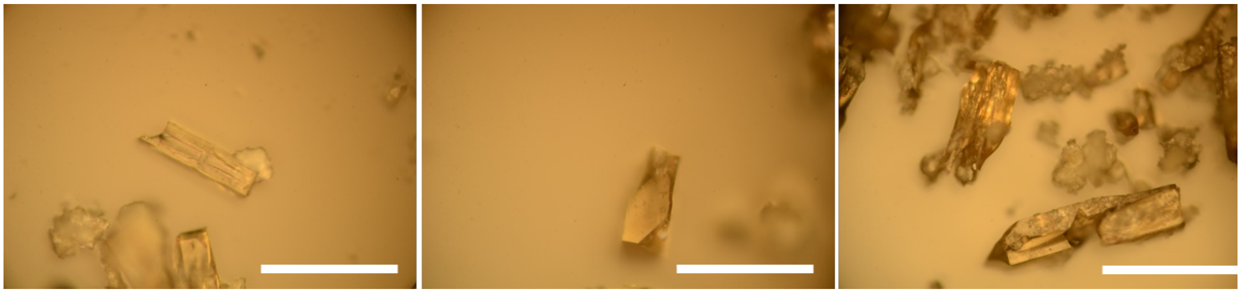

Figure S23. Optical images of different **IMIDFE** crystals. Optical scale bars are 10 µm.

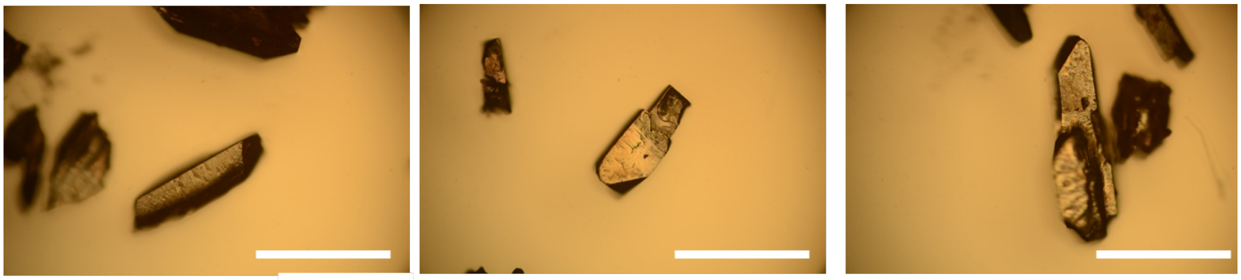

Figure S24. Optical images of different **MUV-24(lla)** crystals. Optical scale bars are 10 µm.

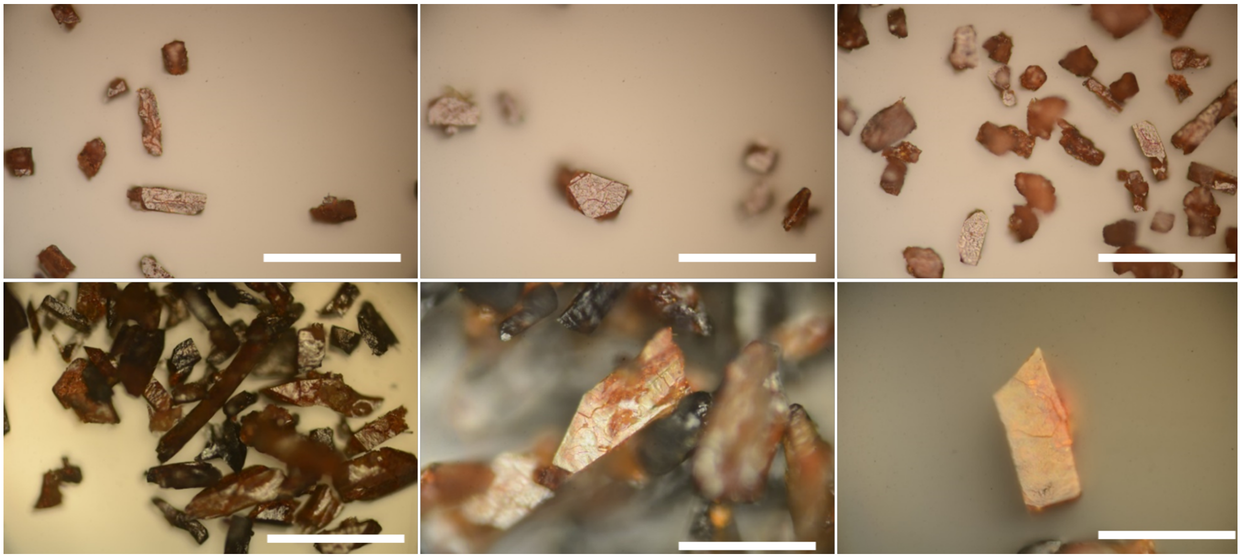

Figure S25. Optical images of different **MUV-24(zni)** crystals. Optical scale bars are 10 µm.

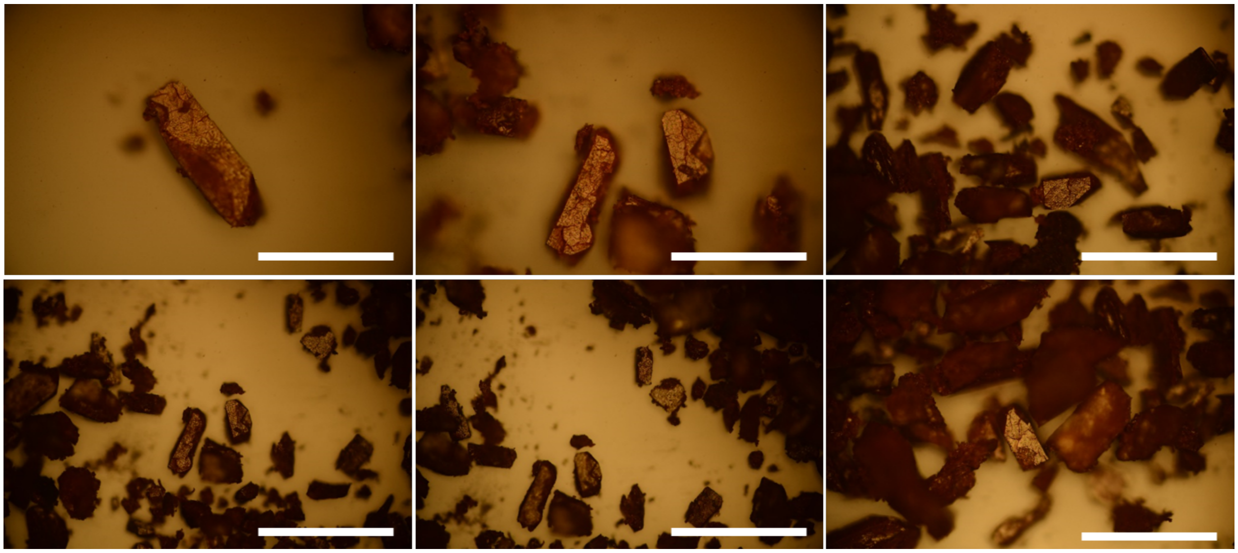

Figure S26. Optical images of different **MUV-24(coi)** crystals. Optical scale bars are 10 µm.

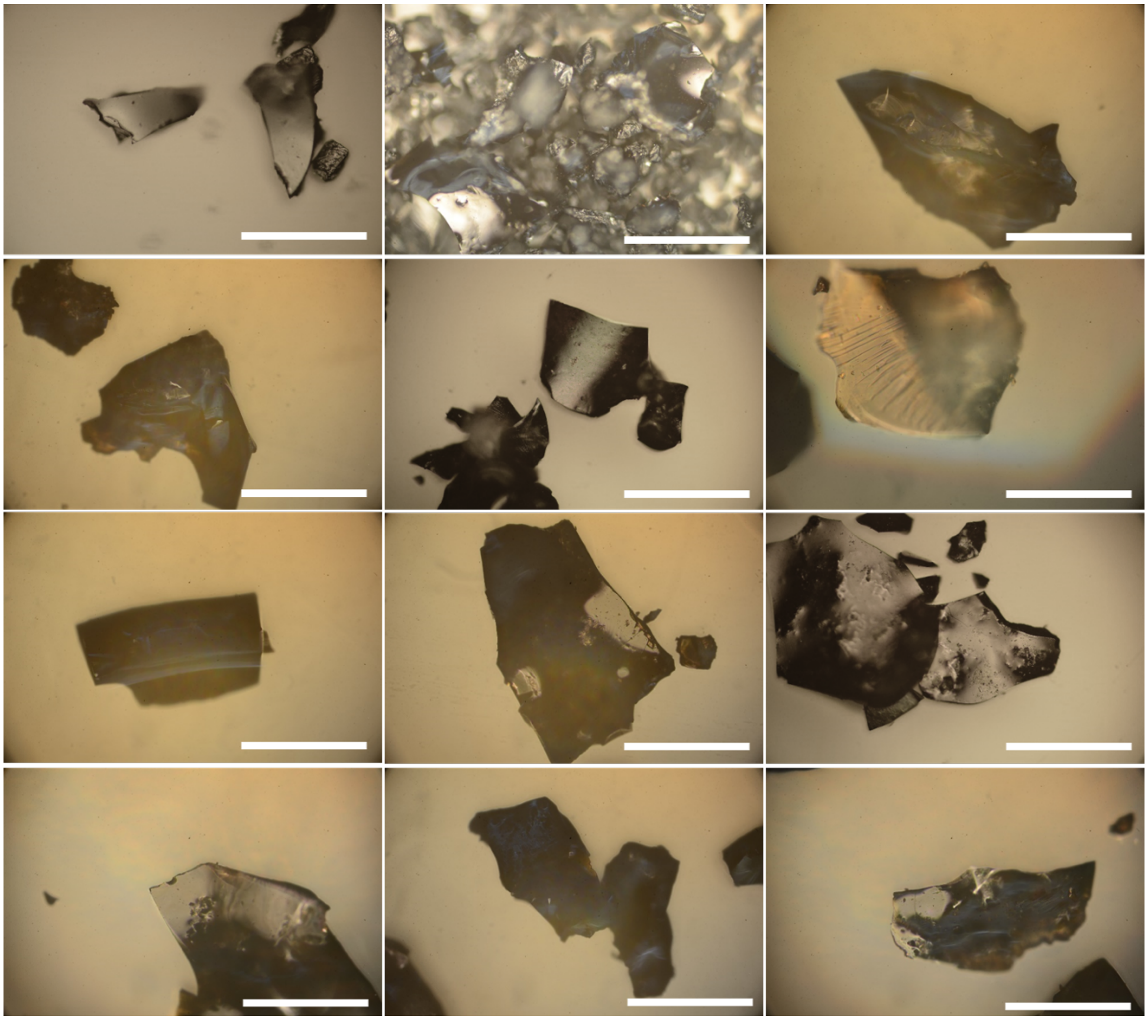

Figure S27. Optical images of different **a_g-MUV-24** monolithic glass. Optical scale bars are 5 µm.

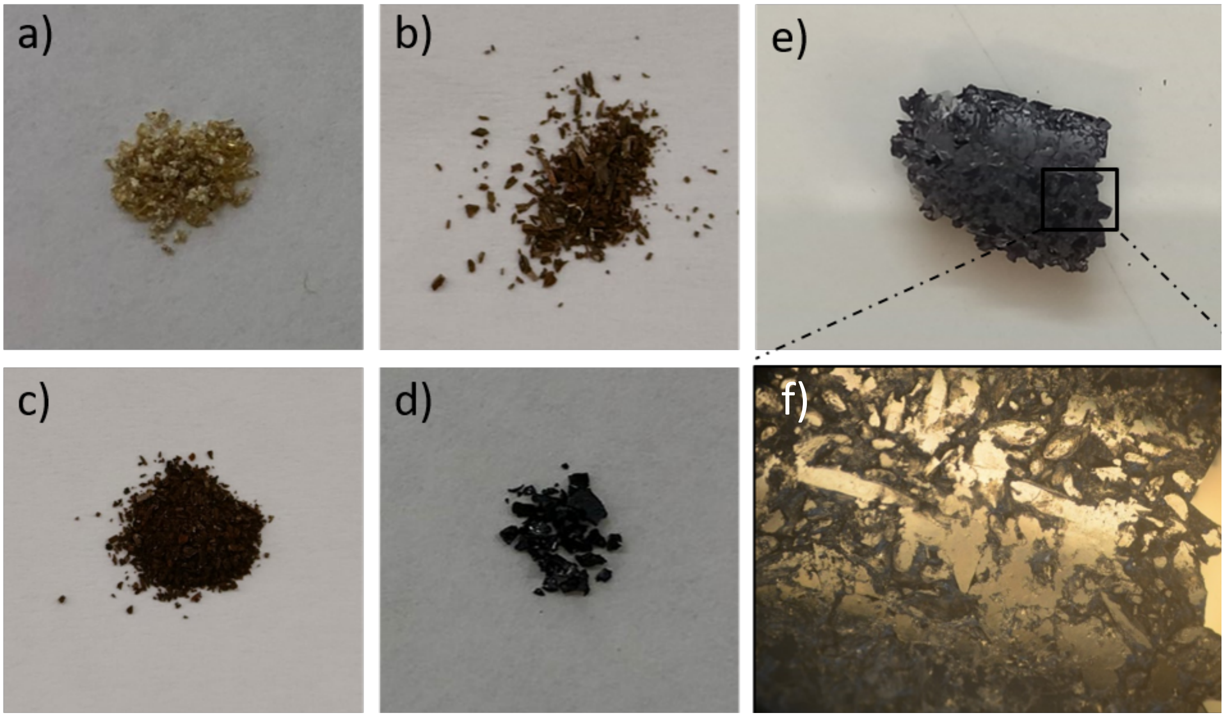

Figure S28. Photographs of different substances obtained after conducting the DSC protocols. a) **IMIDFE**. b) **MUV-24(lla)**. c) **MUV-24(coi)**, these crystals are only identical to those corresponding to the **zni** phase. d) **a$_g$-MUV-24** monolithic, where it is evident that the finely ground ZIF microparticles have been fluxed into a ZIF glass monolith in a melting process. This can be seen more clearly in image e) in which it is observed a 5 mm size monolithic structure formed in the tubular oven, which demonstrates the possibility of obtaining large quantities of this material. f) A zoom of the vitreous structure can be observed.

S10. Nanomechanical properties characterization by Atomic Force Microscopy (AFM)

In this work we performed PeakForce™ Quantitative Nanoscale Mechanical characterization (PF-QNM™) in PeakForce Tapping™ mode, in a Bruker Dimension Icon AFM (Bruker Corporation, CA, USA) to map the topography and the Young's modulus of different materials. PF-QNM permits to record the nanomechanical properties of materials, i.e. modulus, adhesion, dissipation, and deformation, together with their topography. Nanometric and sub-nanometric resolution can be reached in topography as well as in the property channels in a non-destructive approach.

IMIDFE, **a$_g$-MUV-24** and **ZIF-62** (as reference material) were drop-casted on silicon substrates and imaged, in air under ambient conditions, with RTESPA-150 probes (spring constant 5 N/m, Bruker). By means of Peak Force Tapping imaging mode used during the experiments, force-distance curves are performed at every pixel, and they are used to fed to PF-QNM mode (Figure S29). The force applied by the tip was fixed to ~1nN in all experiments. The automatic analysis of these curves generates maps of mechanical properties distribution, and topography simultaneously. Specifically, Young's modulus mapping of the surface is obtained by a Derjaguin, Muller and Toropov (DMT) fitting of part of each force-distance curve (Figure S29).

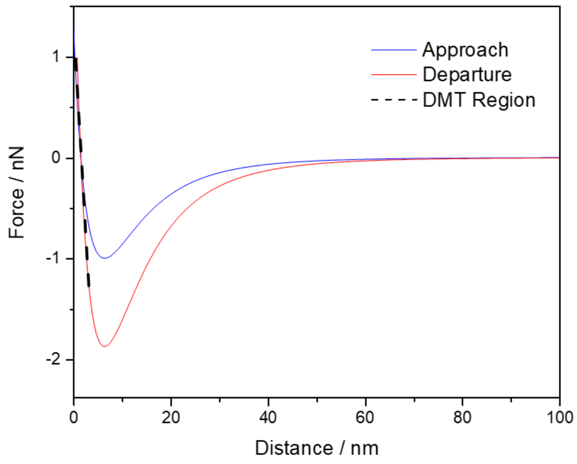

Figure S29. Variation of the force exerted by the tip as a function of the tip-sample distance.

Obtaining reliable values of Young's modulus using this technique requires to be careful during the image acquisition process to avoid damaging the probes. Each probe needs to be characterized for knowing its specific force constant and tip radius. It is worth noting that the tip radius does not remain constant after several measurements. For this reason, it needs to be recalibrated frequently.

In our set of experiments, tip radius was estimated using a Polystyrene reference sample (nominal Young's modulus: 2.7 GPa). The Young's modulus of the reference sample was measured before and

after each sample measurement to guarantee the reliability of the images and the stability of the tip during the process.

Using this method, three set of samples were measured: **a_g-MUV-24, IMIDFE** and **ZIF-62.** The results obtained are depicted below.

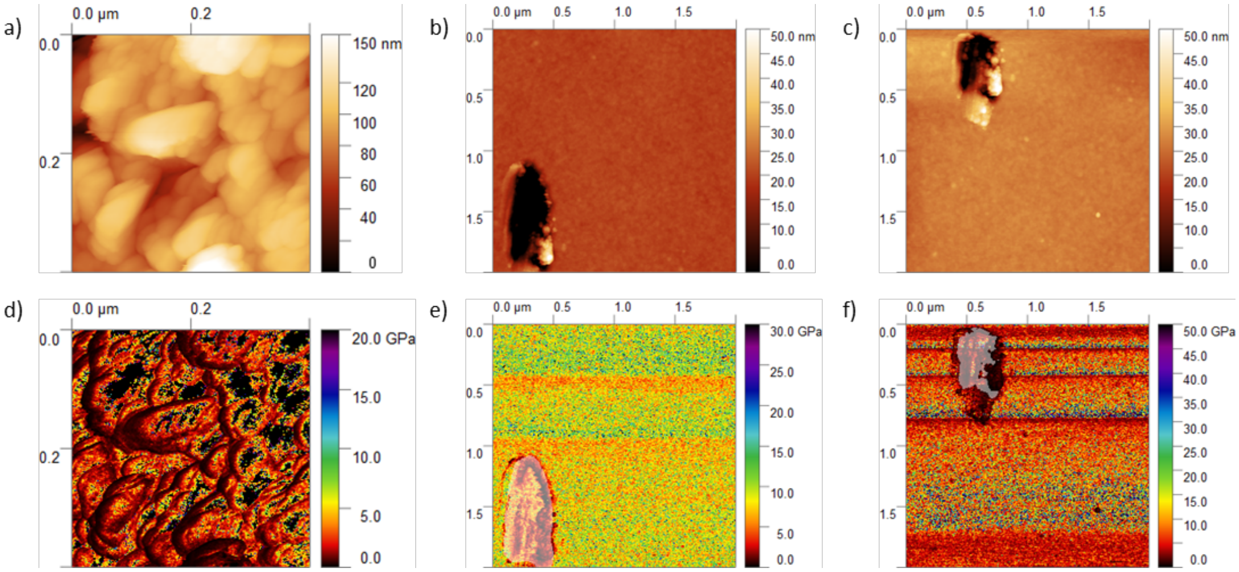

Figure S30. AFM images of **a_g-MUV-24** taken using the method described before. a-c) Topography images of three different samples; d-f) Young's modulus mapping of the same areas as the topography images.

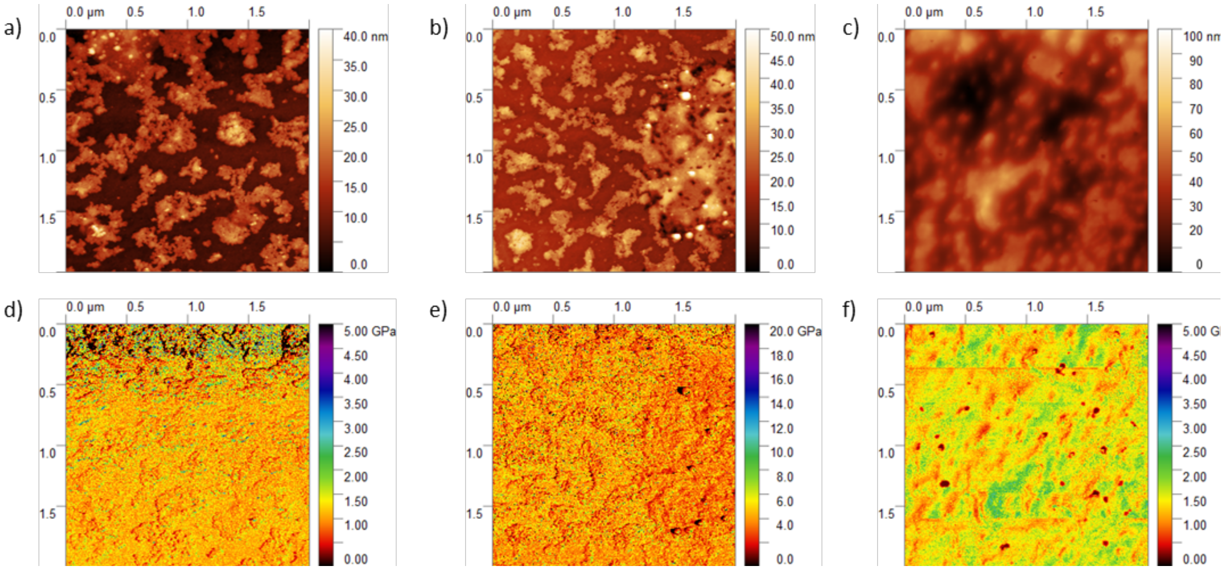

Figure S31. AFM images of **IMIDFE** taken using the method described before. a-c) Topography images of three different samples; d-f) Young's modulus mapping of the same areas as the topography images.

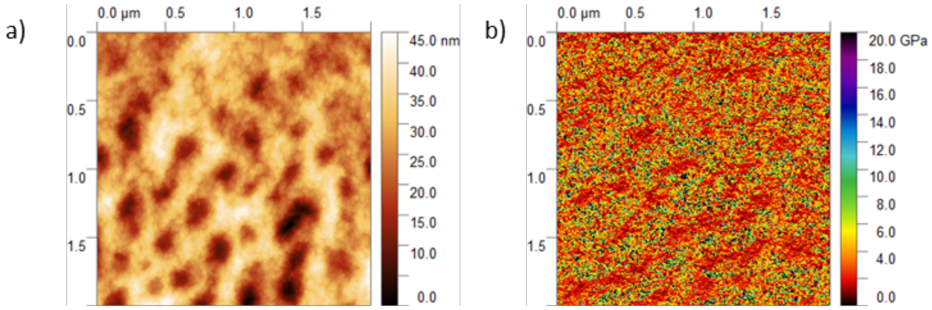

Figure S32. AFM images of **ZIF-62** taken using the method described before. a) Topography image of one area over a crystal; b) Young's modulus mapping of the same area.

Table S2. Young's modulus values obtained for **IMIDFE**, **a_g-MUV-24** and **ZIF-62**. The values correspond to the average of all the pixels of each image taken with a final average of the different images. Standard deviations from the measurements appear in brackets.

	**a_g-MUV-24**	**IMIDFE**	**ZIF-62**
Image 1	10.46 GPa	1.12 GPa	5.98 GPa
Image 2	8.53 GPa	4.17 GPa	
Image 3	10.75 GPa	1.34 GPa	
Average	9.9(±1.2) GPa	2.2(±1.7) GPa	

S11. References

1. G. M. Sheldrick, *Acta. Cryst*., **2015**, *A71*, 3-8.

2. O. V. Dolomanov, L. J. Bourhis, R. J. Gildea, J. A. K. Howard and H. Puschmann, *J. Appl. Cryst*., **2009**, *42*, 339-341.

3. A.K. Soper, Science; Council, T. F., GudrunN and GudrunX: Programs for Correcting Raw Neutron and X-Ray Diffraction Data to Differential Scattering Cross Section, Science & Technology Facilities Council, 2011.
